# Nature Exposure and Its Effects on Immune System Functioning: A Systematic Review

**DOI:** 10.3390/ijerph18041416

**Published:** 2021-02-03

**Authors:** Liisa Andersen, Sus Sola Corazon, Ulrika Karlsson Stigsdotter

**Affiliations:** Department of Geosciences and Natural Resource Management, University of Copenhagen, Rolighedsvej 23, 1958 Frederiksberg C, Denmark; suoe@ign.ku.dk (S.S.C.); uks@ign.ku.dk (U.K.S.)

**Keywords:** BVOCs, forest bathing, green-blue space, human health, immune system, inflammation, inhalation, natural environments, NK cells, terpenes

## Abstract

Given the drastic changes in our lifestyles and ecosystems worldwide, the potential health effects of natural environments have grown into a highly pervasive topic. Recent scientific findings suggest beneficial effects from nature exposure on human immune responses. This review aims at providing a comprehensive overview of literature published on immunomodulatory effects of nature exposure by inhalation of natural substances. A systematic database search was performed in SCOPUS and PubMed. The quality and potential bias of included studies (n = 33) were assessed by applying the EPHPP (Effective Public Health Practice Project) tool for human studies and the ARRIVE (Animal Research: Reporting of In Vivo Experiments) and SYRCLE (Systematic Review Centre for Laboratory Animal Experimentation) tools for animal studies. The synthesis of reviewed studies points to positive effects of nature exposure on immunological health parameters; such as anti-inflammatory, anti-allergic, anti-asthmatic effects or increased NK (natural killer) cell activity. Decreased expression of pro-inflammatory molecules, infiltration of leukocytes and release of cytotoxic mediators are outcomes that may serve as a baseline for further studies. However, partially weak study designs evoked uncertainties about outcome reproducibility and key questions remain open concerning effect sizes, duration of exposure and contributions of specific vegetation or ecosystem types.

## 1. Introduction

During the last century, environmental degradation and urbanisation have caused drastic changes in our lifestyles and living environments [1,2]. Today, more than half of the world’s population live in urban areas [3], and advancements of the digital era have led to a substantial rise in screen time and time spent indoors along with a decline in outdoor activities, especially in the developed world [4]. This has caused a loss of interaction between humans and nature and a progressing feeling of disconnection from the natural world, which can be defined as everything that exists independently of human conduct [5].

The estrangement from nature and other modern lifestyle changes have considerable consequences for human health [6,7]. However, next to being a health resource, the natural environment today also poses substantial risks to human health, not least due to air pollution and contamination of land and water caused by human activity [8]. Toxic pollution ranges among the most prominent environmental health hazards and is responsible for one out of six deaths worldwide [9]; other environmental burdens of disease include exposure to extreme heat, noise, hazardous chemicals, electromagnetic fields and natural disasters [8]. In recent years, negative health effects related to climate change have also been observed [10], especially in urban areas that are particularly at risk of developing urban heat islands (UHI) due to the lack of natural environments. The impacts of the above-mentioned environmental stressors have led to a significant rise of preventable diseases, such as non-communicable diseases (NCDs), which are today the most frequent cause of death worldwide [11]. Globally, more than 20% of all mortalities could be avoided through healthier environments and almost two-thirds of these are related to NCDs [12]. Thus, the relationships between humans, the environment and health are complex and intertwined, and exposure to intact natural environments is connected to better human health on many levels.

A growing body of evidence suggests that various forms of being exposed to nature, such as living close to, frequenting or even looking at environments dominated by living material, are able to provide salutogenic effects on human health [4]. They range from beneficial psychological to physiological outcomes such as attention restoration, improved mood, lowered anxiety and decrease in depressive symptoms, improved cardiovascular, metabolic, oncogenic, respiratory and endocrine function as well as faster healing after surgery and longer life-expectancy [4,13,14,15,16,17,18,19,20]. Often, these benefits are attributed to indirect effects of nature exposure, such as increased physical activity, social interactions, positive mental effects and exposure to sunlight, but recent findings have also highlighted direct physiological mechanisms that are triggered by exposure to natural environments [13,16,17]. This review focuses on direct mechanisms by which nature can affect human health, more specifically on air-borne compounds emitted by natural environments that have the potential to modulate immunological responses when inhaled, such as biogenic volatile organic compounds (BVOCs), terpenes, essential oils, charged ions, pollen, fungi and bacteria.

### 1.1. Nature Exposure and Immune System Functioning

A limited set of studies have pointed to potential immunological benefits from exposure to natural environments [16,21,22]. By boosting immunological defence mechanisms, natural environments might be able to positively influence immunoregulatory pathways [16]. Immunological defence mechanisms are complex, highly specified and tightly regulated processes that fight foreign pathogens by inducing phagocytosis or apoptosis, producing cytokines or antibodies and releasing inflammatory or cytotoxic mediators [21,23]. During a lifespan, successful immune functioning is shaped by microorganisms we encounter in our environments, from other humans and animals, and is then continuously modified by our diets or medicinal use. By being exposed to a broad variety of organisms, the immune system learns to fine-tune the balance between attack and tolerance mechanisms, and is able to develop the regulatory pathways needed to avoid overshooting immune responses to self or harmless allergens [24,25].

### 1.2. Immunoregulation through Biodiversity

Natural environments are able to provide biologically and genetically diverse microbial inputs [26]. Enhanced hygiene, smaller family sizes, increased antibiotic use and lower exposure to food bacteria in today’s industrialised parts of the world increase the likelihood of acquiring an unfavourable microbiota prone to overreact to otherwise harmless organisms [24]. There is robust evidence that a limited gut microbial diversity leads to a higher prevalence of chronic inflammatory conditions such as inflammatory bowel diseases or obesity [24,25], and that reduced contact with “old friends” (bacteria and parasites common in the natural environment) increases the risk of developing asthma, allergies or other hypersensitivity diseases [24,27,28].

Advancing urbanisation and fragmentation of habitats along with the increase of immunological non-communicable diseases in developed countries led to the formulation of *the biodiversity hypothesis* [29]. It is based on the fact that nature is one of the richest sources of microbial input, and that reduced exposure to natural environments and biodiversity may adversely affect our microbiota and its immunomodulatory capacity [24]. The biodiversity encountered in natural environments not only comprises plant, animal, microbial and fungal varieties, but also the genetic variety of those species as well as the variety of ecosystems that serve as their habitats [26,27,29]. Healthy livelihoods depend on such bio-diverse, well-functioning environments being able to provide essential ecosystem services, regulate infectious disease reservoirs and transmission and serve as pool for potential medical treatments, amongst others [26]. Thus, biodiversity loss poses an acute threat to human health.

### 1.3. Immunoregulation through Inhalation of Air-Borne, Volatile Substances

Next to a diverse microbial input, natural environments are also a rich source of airborne substances such as BVOCs that are emitted by above- and below-ground vegetation, rivers and oceans, soils and other natural structures [30]. BVOCs are produced by terrestrial and marine vegetation and make up approximately two thirds of total volatile organic compounds (VOCs) currently emitted in the atmosphere, with forest ecosystems considered the largest emitters of BVOCs [31]. Since their emission is temperature- and light-sensitive, the amount and type of BVOC emitted varies strongly among species, diurnal and seasonal time points and geographic and climatic regions [30]. Next to methane and dimethyl sulphide (DMS) produced by oceanic plankton, the majority of emitted BVOCs belongs to the class of terpenoids [30].

The accredited anti-inflammatory effects of terpenes include both central and peripheral mechanisms. They encompass the reduction of pro-inflammatory cytokines, modulation of oxidative stress and inhibition of tissue infiltration by inflammatory cells, thereby being able to reduce both acute and chronic inflammatory responses in diverse pathological settings [32,33]. Moreover, terpenes can also exert immune-stimulatory effects such as increasing phagocytic activity, enhancing innate immune responses, repressing the expression of certain pro-inflammatory cytokines and increasing immunoglobulin levels [21]. The anti-tumour effects observed are mainly associated with inducing tumour cell apoptosis, inhibiting their proliferation and preventing metastasis [33]. Many of these effects are mediated by essential immunological cellular components, such as natural killer (NK) cells [33].

Besides terpenes, charged ions that occur in the air close to waterbodies might also have beneficial effects on immune functioning, especially in the respiratory tract [34,35]. Water spray is also a source of microbial input [24], and the inhalation of charged ions, airborne microbes and phytoncides emitted by trees is known to affect systemic immune responses in various ways [20].

By removing airborne pollutants, forest ecosystems are also responsible for health benefits resulting from improved air quality. Air pollution is estimated to cause 6.5 million annual premature deaths worldwide already today [9]. Dry deposition of particulate matter (PM) and absorption of gaseous pollutants by leaf stomata is able to remove up to 4 tons of airborne pollution per square mile and year [36]. This impacts acute and chronic immunological mechanisms by protecting against the development of respiratory diseases and significantly lowers mortality rates in the local population [37]. However, trees can also adversely affect air pollution. BVOCs are highly reactive molecules and can form secondary organic aerosols (SOA) with anthropogenic VOCs, thereby producing ozone [30]. SOAs directly affect the climate by scattering incoming solar radiation and acting as cloud condensation nuclei, thereby significantly changing the planet’s radiative balance and potentially leading to a net cooling effect by increasing cloud albedo [30,38]. This increased cloud cover may locally trap pollutants and lead to adverse health effects [15].

Thus, natural environments do not exclusively have beneficial effects on the immune system, but can sometimes even pose a threat to proper immune functioning. A wide range of microorganisms such as pollen grains, fungal spores, mycelium, algae and bacteria are produced by vegetation, especially grasses, and act as potential allergens and might therefore be harmful by causing or exacerbating allergic reactions [20,36]. A growing number of studies have tried to assess the effects of nature exposure on asthma and allergies; however, the overall outcome of these studies is inconsistent and ranges from positive and negative to no associations [13].

### 1.4. Health-Promoting Ecosystem Services and Their Effects on the Immune System

In order to understand the relationship between nature and immunological health in detail and to provide a thorough analysis of its long- and short-term co-benefits and potential adverse effects, it is important to consider the services that ecosystems provide either directly or indirectly for humans to sustain their lives and enhance their wellbeing. Many of these ecosystem services are health-supporting; they provide biodiversity and reduce harmful exposures, e.g. to extreme heat or air and water pollution [36]. The multitude and diversity of human health benefits observed from nature suggest a plurality of mechanisms that either stand side by side or interact in one broad pathway of action [16]. The immune system is a key player in maintaining physiological homeostasis and in sustaining health over disease in the human body. Current literature suggests that enhanced immune functioning can be the outcome of a vast majority of observed nature-related health effects [16]. It has therefore been postulated as a promising candidate that may incorporate many different health effects into one central pathway.

The aim of this review is to provide a comprehensive overview of literature published on the immunomodulatory effects on human health following exposure to natural environments. What distinguishes the paper at hand is its focus on inhalation as the only way of taking in the biogenic substances analysed. The goal was to define a baseline of reliable data that can be used as a starting point for future in-depth immunological research, to shed light on consistencies and potential discrepancies and to elucidate knowledge gaps in this field.

In order to establish a holistic perspective and stimulate a broad interdisciplinary research agenda on immunoregulation through nature exposure, different experimental setups were included in this review. Animal experiments represent an important data source that helps create both initial hypotheses as well as elucidate causal pathways through which nature unfolds its various health benefits. Therefore, both human as well as animal experimental studies were evaluated and rated for their scientific quality.

## 2. Methodology

The methodological approach for the present review followed the guidelines provided by Preferred Reporting Items for Systematic Reviews and Meta-Analyses (PRISMA) [39].

### 2.1. Search Strategy

A structured literature search was carried out in the databases Scopus and PubMed between February and March 2020 and included all articles published up to the search date. The search string was designed to combine different nature-based interventions with different immune-related physiological outcomes (using the Boolean operators AND and OR). A search for article titles including the following keywords was performed:

Nature OR “natural environment” OR forest* OR ecosystem* OR vegetation OR “green infrastructure” OR wood* OR greenness OR greenspace OR outdoor OR biodiversity OR shinrin yoku OR BVOC OR “biogenic volatile organic compound” OR “natural volatile organic compound” OR phytohormon* OR phytoncide* OR “plant gas” OR “essential oil” OR fragrance OR aromatherapy

AND

immun* OR inflamma* OR antiinflamma* OR interleukin* OR cytokin* OR allergen* OR asthma* OR physiologic* OR “NK cell” OR “natural killer“.

When possible, the search was limited to articles and conference papers, and excluded other document types.

The field of immunological health provisioning through nature exposure is multidisciplinary and entails studies with very different methodological approaches which yet have no common narrative, let alone keywords, methodological guidelines or shared objectives. This made it challenging to formulate a fitting keyword search that incorporated all possible wordings and headline formulations into one comprehensive search string and selectively targeted relevant studies in the wide-spanning field. Therefore, we included snowballing as additional search strategy by screening related reviews as well as references of the selected studies, which considerably expanded the results found (see limitations).

### 2.2. Study Selection

Articles retrieved from the database search were roughly screened according to title and abstract for meeting the eligibility criteria, which were defined as follows:

Analysed species were limited to mammals, and ranged from humans (no age or health status restrictions) to animal studies. In vitro studies on cell lines or primary cell material were excluded. A wide range of different nature exposures was considered in the inclusion criteria, such as all kinds of outdoor nature (urban nature, wilderness, green and blue spaces…), particles and gases released or produced by nature (BVOCs, pollen, fungi, moulds…) or man-made nature products (essential oils, fragrances, aromas, wood panels…). Excluded were foods, roots, traditional medicine, drugs and venoms. Only studies with no or light activities were included, since physical exercise is known to have immunological effects in itself [15,16]. Concerning the route of administration, only inhalation or olfactory stimulations were included. This likewise entailed being intentionally exposed to volatile substances in an experimental setting as well as normal breathing of ambient air while being exposed to natural environments, such as forest bathing or “Shinrin Yoku” (Japanese term for “taking in the forest atmosphere” [33,40]). Furthermore included were exposures to specific housing conditions or residential and recreational stays in nature. All other kinds of exposure (like simply viewing nature from inside) or administration routes (such as oral, topic or parenteral) were beyond the scope of this review and therefore not considered. Furthermore excluded were exposures specific to a certain situation or professional context, such as occupational wood dust exposure of forest workers or wood smoke from forest fires or stoves. Studies involving viral diseases and parasite infestations (e.g., Lyme disease, boreliose infections) were also not part of this review.

To be eligible, studies had to examine physiologic parameters attributable to immune system responses like cellular properties, cytokine and antibody levels, or disease-related outcomes serving as indicators for an immune response, e.g. respiratory symptoms, autoimmune conditions or allergic sensitisations. Due to limited resources, the language of included articles was limited to English and the study type to peer-reviewed intervention studies. None of the investigators were contacted, and no unpublished data was retrieved.

### 2.3. Quality Assessment

The included set of articles was divided into human and animal studies, and three distinct but specific quality assessment tools were applied. Each study was independently evaluated by two researchers. In case of disagreements, the individual assessments were discussed and a consensus was found between the researchers.

For human studies, the risk of bias was evaluated following the quality assessment tool developed by the Effective Public Health Practice Project (EPHPP) [41]. Based on six parameters (selection bias, study design, confounders, blinding, data collection methods and withdrawals/dropouts), the studies were rated and classified into overall strong (1), moderate (2) or weak (3). Scoring criteria followed the publicly available EPHPP dictionary. Two or more weak parameters classified a study as overall weakly designed. An overall moderate study may only be rated weak in one parameter, while an overall strong study required no weak parameters.

The quality assessment of animal studies was carried out according to the “Animals in Research: Reporting In Vivo Experiments” (ARRIVE) guidelines for good reporting practice in animal research [42]. These guidelines provide 20 questions concerning the quality of design and reporting in animal research and give a useful indication of the adoption of good scientific practise in animal studies. We rated each question with points from 1 (good/reported) to 3 (weak/not reported).

Additionally, we evaluated the risk of bias of each animal study based on the Systematic Review Centre for Laboratory Animal Experimentation (SYRCLE) tool [43], which was adapted from the Cochrane Collaboration’s Risk of Bias (RoB) tool and developed specifically for the qualitative rating and systematic comparison of experimental animal studies. The SYRCLE tool addresses 10 different domains which are categorised into assessment of selection bias, performance bias, detection bias, attrition bias, reporting bias and other biases. By rating the potential biases of each individual study, we assessed the study to have a low, high or unclear risk of bias.

## 3. Results

### 3.1. Article Selection

**Figure 1:**
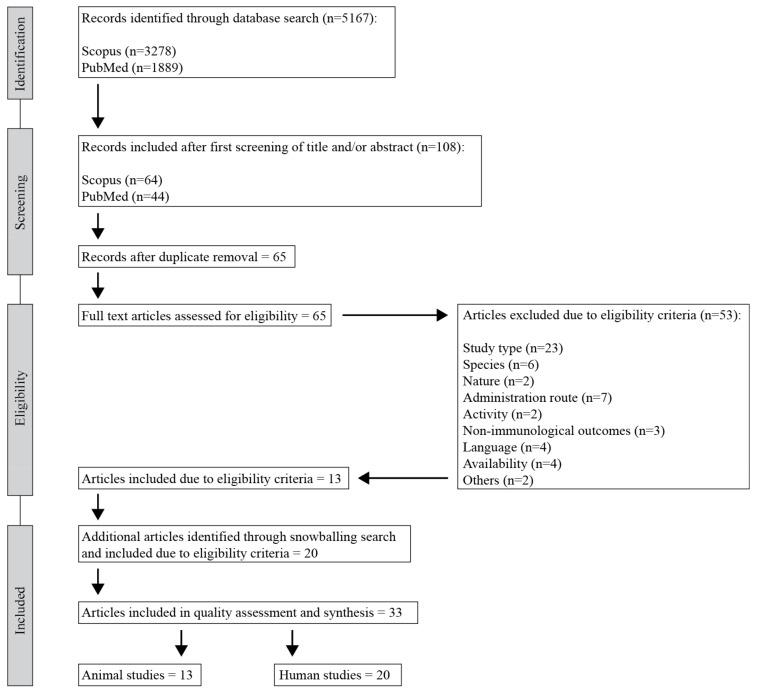
Flowchart of study selection process (following PRISMA guidelines).

The initial database search returned 5167 records (3278 from Scopus and 1889 from PubMed). After a first screening of titles and abstracts and removal of duplicates, 65 studies remained for full-text analysis. Full-text reading resulted in a total of 13 studies that met all inclusion and exclusion criteria. Additional articles were identified through a snowballing search based on scanning references and reviews; 20 of those met the eligibility criteria and were therefore included in the final selection. Summing up, a total of 33 articles were included in this systematic review, comprising both human (n = 20) and animal (n = 13) intervention studies. Figure 1 illustrates the respective stages in the study selection process.

### 3.2. Characteristics of Included Studies

An overview of included human and animal studies is given in Table 1 and Table 2 and shows the year of publication, country of study origin, study design, sample size, sample characteristics, age and sex of sample, type of intervention and control and duration of intervention.

#### 3.2.1. Characteristics of Human Studies

Among the included human studies, only one study was designed as a randomised controlled trial (RCT) [44] and nine studies were controlled clinical trials (CCT) [34,35,45,46,47,48,49,50,51]. The rest were designed as one group pre-post intervention studies [52,53,54,55,56,57,58,59,60,61], three of which were carried out as one group crossover studies [55,59,61]. Sample sizes ranged between 11 and 200 subjects, most studies investigating 10–20 individuals per group. Participant characteristics ranged from healthy individuals to individuals suffering from diverse chronic conditions, and comprised children, adults and elderly people from both genders aged seven to 79 years. The types of interventions could roughly be divided into three experimental setups: forest bathing, experimental inhalation of BVOCs or fragrances and exposure to waterfalls. Forest bathing interventions and waterfall exposures were examined in 14 [44,45,46,47,48,49,50,52,53,54,55,56,57] and two [34,35] studies, respectively. Four studies analysed the effects of BVOC [58] or fragrance [59,60,61] inhalation on human subjects (Table 1).

**Table 1:** Characteristics of included human studies.

Main Author	Year	Country	Study Design	Sample Size (Intervention/Control)	Sample Characteristics	Sample Age	Sample Sex	Intervention	Control	Duration
**Forest bathing**										
Han et al. [45]	2016	South Korea	Pre-post (2 groups) CCT	61 (33/28)	Adults with chronic pain	25–49	Mixed	Forest bathing (pine, oak maple forest)	Normal daily routine	2 days
Im et al. [44]	2016	South Korea	Pre-post (2 groups crossover) RCT	41	Healthy students	18–35	Mixed	Forest environment (pine tree forest)	Urban environment	2 h
Jia et al. [46]	2016	China	Pre-post (2 groups) CCT	18 (10/8)	COPD patients	61–79	Mixed	Forest bathing	Urban stay	3 days
Kim et al. [52]	2015	South Korea	Pre-post (1 group)	11	Adults with breast cancer	25–60	Female	Forest therapy	/	14 days
Li et al. [53]	2007	Japan	Pre-post (1 group)	12	Healthy adults (office workers)	37–55	Male	Forest bathing	/	3 days (2–4 h/day)
Li et al. [55]	2008a	Japan	Pre-post (1 group)	13	Healthy adults (nurses)	25–43	Female	Forest bathing	/	3 days (2–4 h/day)
Li et al. [54]	2008b	Japan	Pre-post (1 group crossover)	12	Healthy adults	35–56	Male	Forest bathing	Urban stay	3 days (2–4 h/day)
Lyu et al. [47]	2019	China	Pre-post (2 groups) CCT	60 (45/15)	Healthy adults	19–24	Male	Forest bathing (bamboo forest)	Urban stay	3 days
Mao et al. [50]	2012a	China	Pre-post (2 groups) CCT	20 (10/10)	Healthy students	20–21	Male	Forest bathing (broad-leaved forest)	Urban stay	2 days
Mao et al. [51]	2012b	China	Pre-post (2 groups) CCT	24 (12/12)	Elderly patients with hypertension	60–75	Mixed	Forest bathing (broad-leaved forest)	Urban stay	7 days
Mao et al. [49]	2017	China	Pre-post (2 groups) CCT	33 (23/10)	Elderly patients with chronic heart failure	66–79	Mixed	Forest bathing (broad-leaved forest)	Urban stay	4 days
Mao et al. [48]	2018	China	Pre-post (2 groups) CCT	20 (10/10)	Elderly patients with chronic heart failure	66–79	Mixed	Second forest bathing trip (broad-leaved forest) after 4 weeks break	Urban stay after previous forest bathing trip 4 weeks ago	4 days
Seo et al. [56]	2015	South Korea	Pre-post (1 group)	21	Children with asthma	7–12	Mixed	Forest bathing (fir tree forest)	/	4 days
Seo et al. [56]	2015	South Korea	Pre-post (1 group)	27	Children with atopic dermatitis	7–12	Mixed	Forest bathing (fir tree forest)	/	4 days
Tsao et al. [57]	2018	Taiwan	Retrospective study (pre-post 2 groups)	200 (90/110)	Healthy adults	34–56	Mixed	Forest workers	Urban residents	1 year
Tsao et al. [57]	2018	Taiwan	Pre-post (1 group)	11	Healthy adults	/	/	Forest bathing (coniferous forest)	/	5 days
**BVOC inhalation**										
Li et al. [58]	2009	Japan	Pre-post (1 group)	12	Healthy adults	37–60	Male	Inhalation of phytoncides (vaporized hinoki cypress stem oil) in urban hotel room	/	3 days
**Fragrance inhalation**										
Kiecolt-Glaser et al. [59]	2008	Ohio, USA	Pre-post (1 group crossover)	56	Healthy adults	18–43	Mixed	Inhalation of fragrances (lavender, lemon)	Inhalation of water vapour	1.25 h
Komori et al. [60]	1995	Japan	Pre-post (2 groups)	20 (12/8)	Adults with depression	26–53	Male	Inhalation of citrus fragrance mix (limonene, citral, other EOs)	*only positive control group*	4–11 weeks
Trellakis et al. [61]	2012	Germany	Pre-post (1 group crossover)	32	Healthy adults	20–45	Mixed	Inhalation of stimulant fragrances (grapefruit, fennel, pepper)	No fragrance exposure	3 days (30 min/day)
								Inhalation of relaxant fragrances (lavender, patchouli, rose)		
**Waterfall exposure**										
Gaisberger et al. [35]	2012	Austria	Pre-post (2 groups) CCT	54 (27/27)	Children with allergic asthma	8–15	Mixed	Waterfall exposure (WF+) in national park	No waterfall exposure (WF-) in national park	3 weeks (1 h/day)
Grafetstätter et al. [34]	2017	Austria	Pre-post (3 groups) CCT	91 (33/32/26)	Adults with stress (Pre-treated with oral cholera vaccination)	19–61	Mixed	Hiking in national park with waterfall exposure (WF+)	Hiking in national park without waterfall exposure (WF-)	1 week (1 h/day)

#### 3.2.2. Characteristics of Animal Studies

The majority of included animal studies were carried out in mice [62,63,64,65,66,67,68,69,70,71] (10 out of 13), while two studies used rats [72,73] and one study worked with guinea pigs [74]. All studies used a pre-treatment such as lipopolysaccharide (LPS), ovalbumin (OVA), Der p (*Dermatophagoides pteronyssinus),* Der f (*Dermatophagoides farina)* or other to experimentally induce an immune reaction which served as control condition for the actual intervention. Sample sizes ranged between 12 and 84 animals; most studies analysed 10 animals per group. Animals were between four and 10 weeks of age and comprised both genders. The types of interventions could be divided into the following experimental setups: inhalation of BVOCs [62,63,64], eucalyptol [65,66,74], limonene [67,68,73], mix of limonene/ozone [69,70], linalool [72] and other fragrances (lemon, oak moss, labdanum and tuberose) [71]. Three studies evaluated the effects of different housing conditions in laboratory animal cages equipped with different wood beddings [62,63,67] (Table 2).

**Table 2:** Characteristics of included animal studies.

Main Author	Year	Country	Study Design	Sample Size (Number per Group)	Sample Characteristics	Sample Age	Sample Sex	Intervention	Control	Duration
**BVOC inhalation**										
Ahn et al. [62]	2018a	South Korea	Animal	35 (7)	Mice Pre-treated with LPS	7 weeks	Male	Housing with BVOC wood panels (C. obtusa, P. densiflora) LPS	Housing without wood panels LPS	4 weeks
Ahn et al. [63]	2018b	South Korea	Animal	49 (7)	Mice Pre-treated with OVA	5 weeks	/	Housing with BVOC wood panels (C. obtusa, P. densiflora, P. koraiensis, L.kaempferi) OVA	Housing without wood panels OVA	27 days
Yang et al. [64]	2015	South Korea	Animal	/	Mice Dinitrochlorbenzene (DNCB)-induced atopic dermatitis (AD)-like disease model	7 weeks	/	Exposure to BVOC (C. obtusa)	Exposure to vehicle	8 weeks
**Eucalyptol inhalation**										
Bastos et al. [74]	2011	Brazil	Animal	ca. 35 (7–10)	Guineau pigs Pre-treated with OVA	/	Male	Eucalyptol (1,8-cineol) inhalation OVA	Saline inhalation OVA	15 min
Kennedy-Feitosa et al. [65]	2019	Brazil	Animal	40 (10)	Mice Pre-exposed to cigarette smoke (CS)		Male	Eucalyptol (1,8-cineol) inhalation CS	Vehicle inhalation CS	120 days (15 min/day)
Lee et al. [66]	2016	South Korea	Animal	/	Mice Pre-sensitised to Der p (house dust mite allergen; HDM)	6 weeks	Female	Eucalyptol (1,8-cineol) inhalation Der p	Vehicle inhalation Der p	/
**Limonene inhalation**										
Bibi et al. [67]	2015	Israel	Animal	30 (10)	Mice Pre-treated with OVA	8 weeks	Female	Housing with Limonene-treated wood bedding OVA	Housing with untreated wood bedding OVA	30 days
Hirota et al. [68]	2012	Japan	Animal	30 (10)	Mice Pre-sensitised to Der f (house dust mite allergen; HDM)	6 weeks	Male	Limonene inhalation Der f	No inhalation Der f	31 days
Keinan et al. [73]	2005	Israel	Animal	40 (10)	Rats Pre-treated with OVA	4 weeks	/	Limonene inhalation (ozone scavenger) Eucalyptol inhalation (inert to ozone) OVA	No inhalation OVA	1 week
**Limonene/ozone inhalation**										
Hansen et al. [69]	2013	Denmark	Animal	ca. 40 (9–10)	Mice Pre-treated with OVA	5-6 weeks	Female	Limonene inhalation Limonene + ozone inhalation OVA	No inhalation Ozone inhalation OVA	14 weeks
Hansen et al. [70]	2016	Denmark	Animal	40 (10)	Mice Pre-treated with OVA	6 weeks	Female	Limonene inhalation	Air inhalation	3 days (60 min/day)
**Linalool inhalation**										
Naka-mura et al. [72]	2009	Japan	Animal	12 (4)	Rats Stressed by restraining in tube	7–8 weeks	Male	Linalool inhalation Stress	No inhalation Stress	2 h
**Fragrance inhalation**										
Fujiwara et al. [71]	1998	Japan	Animal	84 (12)	Mice Stressed with high pressure	8–10 weeks	Male	Fragrance exposure (lemon, oak moss, labdanum, tuberose) Stress	No fragrance exposure Stress	24 h

### 3.3. Quality Assessment

#### 3.3.1. Quality Assessment of Human Studies

For methodological quality assessment, we subjected all included human studies to a risk of bias assessment following the EPHPP quality assessment tool [41]. 

According to the EPHPP tool, one study was rated overall strong [61], four studies as overall moderate [48,49,52,56] and 15 studies got a weak overall score [34,35,44,45,46,47,50,51,52,53,54,55,57,58,59,60] (Table 3). The weak ratings were mainly due to lack of information on recruitment procedures, the use of self-referred or non-representative samples (selection bias) and missing information on blinding. Blinding was neither described for study assessors nor for participating subjects, the latter being hardly applicable in multi-day forest bathing studies where the exposure to environmental surroundings is obvious. In order to more representatively evaluate the methodological quality of included studies, we therefore chose to also provide an alternative overall score excluding the “blinding” parameter. This resulted in five studies being rated as overall strong, nine as moderate and six as weak (Table 3, last column). More information on scoring criteria is given in the Supplementary Material.

#### 3.3.2. Quality Assessment of Animal Studies

All animal studies were evaluated following two standardised quality assessment guidelines: ARRIVE assessment tool [42] and SYRCLE’s risk of bias tool for animal studies [43]. The results from the ARRIVE assessment, entailing 20 categories, are summarised in Table 4. Specific information on assessment criteria for individual ratings are provided in the Supplementary Material.

Most studies provided a good to moderate title, abstract and introduction section. In the methods section, most studies met the criteria concerning the description of experimental procedures, animal details and housing and husbandry conditions (categories 7–9), while no study appropriately provided a calculation of sample sizes (category 10) or described the method of allocation to experimental groups (category 11). Almost all studies provided sufficient information regarding the experimental outcomes (category 12) and included a description of the statistical analysis of the results (category 13).

In the results section, we detected more reporting shortcomings than in the other categories. A failure in reporting group-specific baseline data of experimental animal characteristics (category 14) as well as reporting adverse events (category 17) was asserted in almost all studies. The majority of studies failed to monitor and report specific baseline characteristics such as body weight. Moreover, many studies did not report any information on the numbers of animals included in the final analysis (category 15), nor refer to drop out rates or reasons for exclusions. All studies included a measure of precision (e.g. bars representing standard deviations) in their outcome report (category 16), but only one study [69] also provided data on the number of individual data points within one group, which should be considered the gold standard of outcome reporting.

Regarding the discussion section, most studies interpreted the implications of their findings within the current scientific literature; however many failed to elaborate on study limitations (category 18) and generalisability of study outcomes (category 19), i.e. translation of outcomes into the human system.

A risk of bias assessment following the SYRCLE’s risk of bias (RoB) tool for animal studies [43] was conducted to complement the ARRIVE quality assessment. According to the SYRCLE RoB tool, many studies did not adequately report measures taken to reduce potential risk of biases in several categories. Randomisation and blinding of the experimental setup were rarely reported, and no study had performed a preceding calculation of sample sizes. All results of the SYRCLE risk of bias assessment are provided in the Supplementary Material (Table S1).

### 3.4. Outcomes and Synthesis

#### 3.4.1. Outcomes of Human Studies

**Figure 2:**
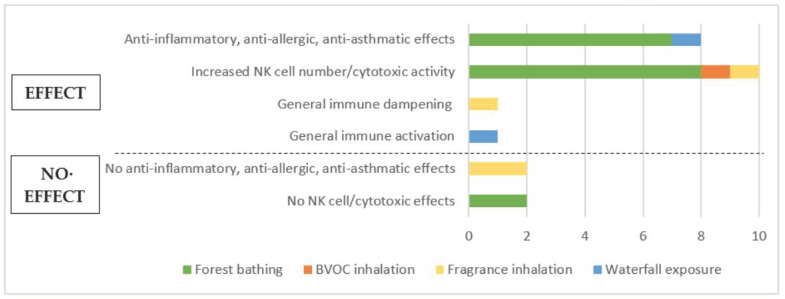
Overview of outcomes from human studies.

The majority of studies measured either anti-inflammatory or cytotoxic effects following nature exposure. Experimental parameters analysed were mainly expression of pro- or anti-inflammatory cytokines (mostly IL-6 and TNFα) in serum, numbers and percentages of immune cell subsets (mostly NK cells and T cells) and expression of cytotoxic mediators (perforin, granzyme A/B and granulysin) as well as cytotoxic NK cell activity. Some studies examined several outcomes. Overall, a positive effect was observed on most immunological parameters measured; four studies showed no significant changes. An overview of outcomes measured in studies with human subjects is presented in Figure 2.

Most studies with human subjects examined the effects of forest bathing trips of varying lengths on different immunological parameters. Seven (out of 14) forest bathing studies analysed inflammatory cytokine expression and unanimously reported either a decrease in pro-inflammatory and/or an increase in anti-inflammatory cytokine levels [44,46,48,49,50,51,56], implicating anti-allergic or anti-asthmatic outcomes from being exposed to forest environments. One of the studies also evaluated changes in clinical scores of patients with asthma and atopic dermatitis after a forest trip and concluded a relief of clinical symptoms and beneficial effects of forest bathing on spirometric outcomes [56].

Ten (out of 14) forest bathing studies elucidated the effects on the distribution of immune cell subsets as well as on their distinct effector activities, with a special focus on NK cells. The majority of these studies (8 out of 10) reported an increase either in NK cell number or in NK cell activity [45,47,52,53,54,55,57], which was measured either directly by flow cytometry or indirectly by assessing the level of cytotoxic mediators circulating in the blood. Two forest bathing studies did not observe any significant changes in NK cell outcomes [46,50]. One additional study reported an increase in NK cell number and activity after the isolated inhalation of phytoncides under laboratory conditions [58], concluding that volatile substances released by plants are responsible for the effects observed in natural environments.

Three studies examined the outcomes of inhaling different fragrances (citrus mix, lavender, lemon, grapefruit, fennel, pepper, patchouli and rose) on immunological parameters such as cytokine and chemokine levels, cell ratios and strength of immune response to infection. Two of these studies reported no significant effect of fragrance exposure on levels of circulating cytokines and chemokines [59,61], while one of them observed a lower hypersensitivity to candida infection compared to control [59]. Another study reported beneficial effects on immune cell ratios after fragrance exposure [60].

Two studies looked at potential health effects of charged ions in ambient air in the vicinity of waterfalls. One of them detected significantly decreased pro-inflammatory cytokine levels combined with enhanced lung function and reduced clinical symptoms in children with allergic asthma, which was suggested to be due to an induction of circulating regulatory T cells [35]. The other study reported that exposure to waterfalls led to an activated immune system and also improved lung function [34].

Table 5 provides a comprehensive summary of all outcomes from human studies.

#### 3.4.2. Synthesis of Human Studies

The synthesis of human studies analysing the immunological effects of forest bathing points to largely positive evidence of anti-inflammatory and anti-asthmatic effects along with a promising evidence of enhanced cytotoxicity stemming from increased NK cell levels or activities. However, the synthesis of anti-inflammatory effects was obscured by differing results derived from analysing cytokine levels, specifically IL-6 and TNFα. Some studies only observed an alteration in one of the cytokines and no change in the other, while other studies reported a change in the respective other cytokine value. Since the results reveal overall anti-inflammatory effects, the evidence base is still regarded as largely positive, but not entirely conclusive. Several forest bathing studies also measured immune cell subset distributions. While no significant changes in T cell numbers and/or percentages were observed, most studies showed an increase of NK cell levels along with elevated percentages of cells with cytotoxic content (perforin, granzyme A/B and granulysin).

Studies without control groups and/or without sufficient baseline values to adequately control for confounders were heavily represented among forest bathing studies. Almost half of the studies were carried out as one group pre-post intervention designs [52,53,54,55,56,57]; therefore, their findings have less strength but are backed up by similar results from the included CCT studies. Furthermore, a considerable number of forest bathing studies did not provide a thorough calculation of significances for certain outcome measures that were nevertheless interpreted by the study authors as showing an effect. Moreover, most studies comprised a low number of study participants (<30 subjects), which weakened the evidence base for the measured effects significantly. Only four forest bathing studies analysed larger group sizes [44,45,47,57]. One of them showed retrospective results from a big cohort of subjects living in a forest environment, but lacked baseline values of respective measures [57]. Three other studies with larger sample sizes either provided no pre-intervention data [44], no appropriate significance calculations [47] or ignored considerable baseline differences [45], which weakened their results. The findings on NK cell distributions were underlined by positive results from one laboratory experiment simulating BVOC exposure in natural environments [58]; however, this study provided no control group and must therefore be interpreted cautiously. Overall, the evidence base for the effects of forest bathing on immune functioning can be regarded as promising, despite substantial study design shortcomings.

The three studies analysing fragrance inhalation showed highly heterogeneous results that ranged from beneficial immune responses to no measurable effects at all. The different study designs and measured parameters resulted in differing outcomes, which barely suffice to formulate overall tendencies. It is noteworthy that the two studies describing certain positive effects were relatively old [59,60] and one of them used out-dated methods (for details, see Supplementary Material) [59]; accordingly, these results must be questioned in terms of reliability and validity.

Lastly, the two studies analysing the effects of waterfall exposure on immunological health provided good study designs with appropriate controls and group sizes as well as methodologically transparent approaches [34,35]. Thus, their results showing anti-inflammatory and anti-allergic outcomes as well as beneficial clinical outcomes provided a reliable evidence base which remains to be confirmed by a greater number of studies.

In conclusion, human studies provide a promising evidence base for immunomodulatory effects following exposure to natural environments, though general shortcomings in the study designs weaken their soundness.

### 3.5. Outcomes and Synthesis of Animal Studies

#### 3.5.1. Outcomes of Animal Studies

**Figure 3:**
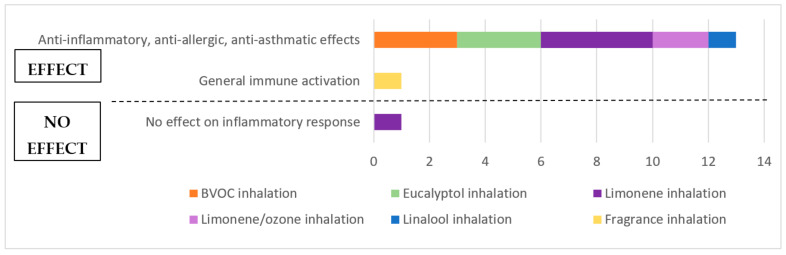
Overview of outcomes from animal studies.

Most animal studies examined the effects of phytoncide or aroma inhalation on a pre-induced immune response. Experimental parameters analysed were levels of various cytokines and/or antibodies in sera and/or bronchoalveolar lavage fluids (BALF) of the respiratory tract, leukocyte numbers (mostly neutrophils, eosinophils, macrophages and lymphocytes) as well as histological or clinical symptoms such as cellular or structural changes, inflammatory cell infiltrations into lung tissue or respiratory markers. Some studies assessed several outcome measures. Overall, a positive effect was observed on most immunological parameters and only one study showed no significant changes. An overview of outcomes from animal studies is presented in Figure 3.

By measuring a decrease in pro-inflammatory and/or an increase in anti-inflammatory (IL-10) cytokines as well as a reduced number of leukocytes, 13 animal studies reported anti-inflammatory, anti-allergic and/or anti-asthmatic effects following exposure to natural substances. All studies analysing BVOC [62,64,75] and eucalyptol [65,66,74] inhalation attested to protective and therapeutic anti-inflammatory effects of these substances in asthma- or allergy-challenged animals. The results for limonene were more heterogeneous, with four out of five studies observing potentially beneficial, anti-inflammatory effects in challenged animals [67,68,70,73], while one study showed no significant effects of limonene inhalation [69]. The mixture of limonene and ozone inhalation was able to protect from the adverse effects elicited by inhalation of only ozone in two studies [69,70]. Furthermore, inhalation of linalool was able to change the immune cell distribution of previously stressed rats, as one study reported [72]. Another study observed that exposure to certain fragrances was able to induce a general immune activation, which they diagnosed by measuring the number of plaque-forming cells in spleen and the thymic weight [71].

The findings of all animal studies are summarised in Table 6.

#### 3.5.2. Synthesis of Animal Studies

Overall, animal studies point to largely coherent and unambiguous evidence for anti-inflammatory effects of phytoncide inhalation in immune-challenged animals. Most experimental setups could easily be compared to each other, since they were relatively homogeneous concerning their methodological approaches, sample sizes and outcome measures. Many studies examined a broad range of inflammatory cytokines and specific antibodies, some measuring both mRNA expression and protein levels. All studies reported widely concordant outcomes, as almost all interventions were able to reverse the inflammation induced by the distinct pre-treatments. Differences were found in the statistical analysis and evaluation of results, with some studies providing rigorous statistical significance calculations and others none. Especially the studies examining BVOC and eucalyptol inhalation comprised a good study design and statistical evaluation, rendering strong evidence for the beneficial effects of these treatments.

Out of the three studies examining limonene inhalation alone, two lacked significance calculations [67,73] while one provided good statistical evaluations [68]. This made it difficult to reliably conclude on the effects observed from limonene inhalation, but points to a positive immune response derived from these experiments.

In contrast, two studies analysing the combined effects of limonene and ozone inhalation in pre-sensitised animals failed to provide essential baseline values before sensitisation, did not show any sensitisation-only control values and lacked significances and statistical comparisons between relevant groups [69,70]. These extensive shortcomings made it impossible to draw any conclusions based on these experiments.

Lastly, single experiments measured the effects of linalool [72] and fragrance [71] inhalation, respectively. One of them used out-dated methods (see Supplementary Material for further details), which made the results less robust [71].

In conclusion, animal studies included in this review provide a solid evidence base for anti-inflammatory, anti-asthmatic and anti-allergic effects upon inhalation of nature-derived substances, especially BVOCs and eucalyptol.

## 4. Limitations of the Review

Limitations are found in the study selection process, which might be biased due to the interdisciplinarity of the topic and the difficulties generated in performing a fitting keyword search. Studies in the field of immunological health and nature have yet no common narrative, making it difficult to formulate a search string that selectively targeted studies from different disciplines. Also the variety of interventions and methodological approaches made it challenging to incorporate all possible wordings into one comprehensive search string. Especially within the field of animal studies, many studies focused on the analysis of a single substance and only included this specific term in their titles and keywords. This problem was handled by including snowballing as additional search strategy. Nevertheless, it cannot be ruled out that individual studies which would also meet this review’s inclusion criteria are missing from the present review. Furthermore, due to the large number of articles resulting from the initial keyword search, it was not possible to include more than two databases in the search. However, included databases gave entirely overlapping outputs, with Scopus being the database having most relevant hits.

Association studies were excluded from this review, since they require different quality assessment approaches and a separate synthesis, which was beyond the scope of the review. Nevertheless, the field provides a high amount of large and rigorously designed population-based association studies that render compelling insights into and provide supplementary evidence for nature’s effect on the immune system. It therefore remains an open task to systematically assess association studies published on this topic and evaluate their outcomes, which could subsequently be merged with the final synthesis of the review at hand.

## 5. Discussion

Until recently, scientific studies on nature and human health have largely been separated into traditional research fields such as environmental science, ecology, biology, geography, landscape architecture, medicine, psychology, epidemiology and public health. This has generated an impressive amount of data that is now starting to be fruitfully brought together in a holistic perspective to stimulate a broad, interdisciplinary research agenda on environmental health. A considerable number of reviews have emerged that illuminate connections between nature and many human health challenges [11,76,77,78]; however, studies focusing on immunological health benefits have so far been underrepresented. This review examined both human and animal studies and found a promising evidence base for immunomodulatory effects following exposure to natural volatile substances or environments, comprising anti-inflammatory, anti-asthmatic, anti-allergic and cytotoxic responses from inhalation of diverse nature-derived compounds.

Whether or not natural environments have the potential to alleviate or even prevent immunological health problems remains an open question that needs more investigation from a multi-dimensional perspective. Animal studies are a strong tool to formulate a robust initial starting point and can be used to back up findings from studies with human subjects. In this review they represent an important data source concerning changes in expression levels of immunological key molecules resulting from the inhalation of biogenic substances. They are used to dissect mRNA and protein expression of inflammatory molecules in various tissues (lung, bronchioles, skin) and not only in blood, as it is commonly done in human experiments. The advantage of animal experiments is that they can easily screen a set of potential hypotheses with relatively low effort and be carried out in standardised, controllable conditions. Unequivocally, translating results into the human setting is challenging, since the standardised experimental setups do not correspond to the multi-faceted aspects of human life. Further, the ethical aspects of using experimental animals need to be diligently balanced with the scientific gains and alternatives considered whenever possible. At present, laboratory experiments are fundamental in disentangling mechanistic pathways and establishing a data base that can eventually be tested in the human system. The positive evidence base derived from animal experiments included in the present review supports the notion that immune functioning might represent a direct, central pathway of how nature and health are connected.

### 5.1. A Baseline for Future Research

Next to providing a comprehensive literature overview, the goal of this review was to define a baseline of existing data for future research. However, since most included studies diverged in their methodological approaches and only performed a superficial screening of varying parameters, this baseline cannot be explicitly defined. Animal and human experiments analysed largely distinct parameters. While animal studies screened a wide range of different cytokines along with measuring detailed leukocyte subset distributions, most human studies did not provide such a thorough analysis of inflammatory parameters and focused more on specific cytokines and cytotoxic mediators. Thus, the human study outcomes are relatively fragmented and lack a comprehensive insight into the distribution of other immune cell types and properties. Therefore, data derived from included human and animal studies in this review can hardly be linked. Nevertheless, a clear anti-inflammatory and cytotoxic tendency can be observed in the majority of studies. Decreased expression levels of many pro-inflammatory molecules in various tissue and blood samples along with an infiltration of leukocyte subsets and an increase of NK cell activity and release of cytotoxic granules are results that may serve as a baseline for further studies.

### 5.2. Study Shortcomings and Recommendations for Future Study Designs

Overall, the synthesis of study findings carried out in this review presents some promising evidence for the positive influence of nature exposure on various aspects of immune functioning. However, considerable shortcomings in the design and conduct especially of included human studies weaken their solidity. Most studies examined only one independent replicate (trials were carried out once), leaving the study inherently prone to random and systematic errors that can only be ruled out by trial repetitions (at least three independent replicates). Moreover, the high number of human studies completely lacking controls gives rise to major concerns regarding the reproducibility and reliability of study outcomes. It has been shown that “within-subject” studies are susceptible to bias since an individual’s initial physiological outcome value can influence the extent and direction of post-intervention responses [79]. Furthermore, most studies neither monitored environmental conditions nor adjusted for potential changes in airborne parameters such as temperature, humidity or BVOC concentrations, which makes it hard to account for individual parameters that might influence study outcomes. The majority of animal studies did not show comparable shortcomings; however, selected studies lacked important baseline values as well as statistical comparisons between relevant groups. In general, included animal trials were also not repeated to generate three independent replicates, which again represents a main drawback concerning the solidity of outcomes.

Ideally, future studies should encompass relatable animal and human experiments including sufficient and adequate controls (placebo controls, controls of external conditions as well as positive and negative control groups). Moreover, they should calculate effect sizes and provide dose-response relationships. In order to guarantee outcome reproducibility, results should be consolidated by a minimum of three trial repetitions. Along with improving the rigor of study designs, the study field should be expanded to bigger group sizes and diverse environments and use more in-depth, state-of-the art analytical methods such as next generation sequencing and big data technologies.

### 5.3. What Is the Optimal Type, Length, Season and Dosage of Nature Exposure?

Studies included in this review originate from different research fields and approach the main question from distinct scientific angles. Although the included studies cover different target groups, exposure types and durations, some essential questions concerning the maximal efficacy of nature exposure still remain open. Which type of nature is best suited for governing positive immunoregulatory effects? How long should nature exposure last and which dosage do we need to ensure a prolonged, but safe, effect? When is the optimal timeframe for nature contact in order to gain the maximum effect?

A factor that might influence the efficacy and magnitude of immune response to natural environments is the type and variability of vegetation that humans are exposed to, also termed eco- or geodiversity [20]. This encompasses both diverse landscape types such as forests, meadows, mountains, coastal areas or oceans as well as the specific species that can be found in these habitats. Human studies in this review mainly analyse forest environments, but encompass a range of different forest types such as broad-leaved, coniferous or bamboo forests. Animal studies also highlight immunomodulatory properties of different BVOCs and natural fragrances, but fail to relate them to specific vegetation types in the natural world. Therefore, it cannot be concluded from the studies included here which type of nature is best at conferring immunological benefits. However, catalogues of different species’ BVOC emission rates from needles and leaves are available [80,81]. BVOC emission potentials depend on environmental factors such as temperature and light, and species-specific factors such as plant age, developmental stage, intercellular CO_2_ concentration, stomatal conductance, leaf structure and gas storage potential [31,82]. Seasonal and diurnal variability have also been observed, and summer seemed to be the best time for using forest environments for medical purposes due to highest temperatures and best light conditions [31,82]. Preliminary evidence points to a characteristic daily emission pattern which might at least apply on clear and calm days, while cloudy and windy conditions appear to be least beneficial for the uptake of BVOCs from forest environments [31,82], but further research is necessary to link these findings with policies that promote public health gains.

Concerning the duration of exposure, this review included studies with exposures ranging from a few hours to several days and weeks. Animal studies were especially useful to elucidate longer exposures of up to four months. However, no conclusion on minimal durations or dosage of nature exposure can be drawn due to the various experimental setups and substances tested. Along with the missing calculation of effect sizes, exposure characteristics need to be analysed in much more detail in order to correctly interpret the study results. Very limited research has so far been carried out to address the question of explicit exposure-response relationships between nature and human health, and no studies included in this review provided relevant answers on this topic. However, some large-scale, population-based research has tried to establish an association between the duration of nature exposure and observed health effects. One study showed that spending a minimum of two hours a week in nature improved overall health and wellbeing, with positive associations peaking at approximately four hours of exposure [83]. Another study examining the associations between frequency, duration and intensity of nature exposure observed a reduction in the prevalence of depression and high blood pressure following nature visits longer than 30 min a week [84]. However, these studies can only provide fractional answers and do not address immunological responses at all.

Another interesting question relates to the optimal exposure time point in life. The review at hand includes studies with adults, elderly and children, but none of the studies compared different exposure timeframes in different target groups; thus, this question cannot be answered here. However, association studies provide evidence for optimal time windows of nature exposure concerning immunological effects. Some studies show that very early exposures during pregnancy and childhood exhibit a greater immunomodulatory effect than exposures later in life [85,86]. Other studies report that exposures during late childhood up to the age of 10–15 years are associated with a lower risk of developing multiple sclerosis [87,88]. However, it seems that later exposures during adult life are also beneficial for certain immune parameters such as the immunoregulatory capacity of helminth infections [89]. To gain profound knowledge on the optimal exposure time point in life, longitudinal research monitoring immunological effects over longer timespans in different target groups is needed.

### 5.4. Understanding Biomedical Mechanisms

A wide range of studies exists that describes immunomodulatory effects of natural substances in vitro or in vivo by other administrative pathways than mere inhalation. Recently, the main molecular targets of terpenes in inflammatory diseases have been summarised and categorised into six groups [22]: inflammatory mediators (interleukins, TNFα, NO and COX2), transcription factors (NF-κB, Nrf2), signal transduction molecules (MAPK signalling molecules such as ERK, p38, JNK, but also STAT3, TRPVs, CB(2)R), oxidative stress (ROS, H_2_O_2_) and autophagy (by targeting apoptotic genes). Disentangling key molecular components and action pathways provides important information for understanding the biomedical mechanisms of how nature affects the immune system, which is essential for its effective and targeted application. However, most studies used parenteral, oral or topical administration, which possibly increases effect sizes compared to studies set in natural environments. Therefore, it remains an open task to verify this evidence in more natural interventions that mimic real life exposures. The review at hand focuses on studies that only use inhalation of volatile substances as administrative pathway. Advantages of this approach are a low implementation threshold and a relatively easy transferability into therapy and policy measures. However, smaller and less coherent outcomes stemming from experiments performed in natural environments should not be confused with evidence obtained from invasive administrations. The challenge of the former will be to define effect sizes and molecular action pathways as clearly and in as detailed a manner as in invasive drug studies.

### 5.5. Nature-Based Clinical Applications

Considering the wide range of potential health benefits that nature provides, possibilities of how to effectively harvest these natural goods for future therapeutic applications can be envisioned. Owing to the rapid rise of diseases related to misled immunoregulation and the frequent severe side effects of currently used immunomodulatory drugs [90,91], there is a growing need for discovering new therapeutic options that are better tolerated. Nature-based interventions might represent such an option; however, potential adverse effects need to be considered when using nature in therapeutic and preventive clinical applications or implementing it in policy recommendations. Many terpenes are not harmful themselves, but can easily oxidise upon air exposure and create allergenic or inflammatory secondary molecules. One study reported irritations of the respiratory tract in mice after exposure to oxidation products from α-pinene and d-limonene [92]. Another study analysing the association between indoor VOCs and lung function reported α-pinene as one of 10 substances negatively influencing human lung function [93]. Auto-oxidation of various terpenes such as α- and β-pinene, limonene, camphor and β-phellandrene has also been observed to have negative allergenic effects on atopic dermatitis [94,95,96]. Thus, the observed adverse effects are concentration- and exposure-dependent, and call for a detailed evaluation of the safe concentration range of terpenes. Furthermore, the use of terpenes through “milder” exposure routes such as forest bathing or nature trips may constitute a potentially safer therapeutic strategy than their direct intake or skin application. Considering the small amount of data that exists on the potential adverse effects of volatile biogenic substances, it is yet too early to draw any conclusion from these studies. Nevertheless, the synthesis of the studies included in this review supports the notion that breathing in nature-derived compounds is overall beneficial for reducing inflammation and promoting immune homeostasis.

## 6. Outlook

Besides the possible clinical use of nature, the findings of the studies in this review also point to potential benefits from promoting nature in official policy frameworks. Inhaling substances emitted from trees in close living proximity or being exposed to wood in daily housing environments display promising examples of co-benefits derived from sustainable developments tailored to support public health gains [13,36]. Possible policy measures could be to promote the conservation of natural environments, enforce the construction of houses from natural materials such as wood and enhance green infrastructure in urban regions. Green infrastructure could be a very effective and multifunctional measure to mitigate negative health impacts e.g., from urban heat islands in cities [36]. It may comprise vegetation planted in urban areas but also engineered structures that fulfil specific functions such as sustainable urban drainage systems (SUDS) [97], and may range from street trees, green roofs and walls, parks, allotment gardens to rain gardens and water basins. Pioneering policy recommendations have defined nature as a cost-effective planning tool for healthy cities [98]. Exposure to nature may therefore be an option to address a range of health challenges and be most effective if designed to harvest both direct as well as indirect benefits from nature (such as physical activity, social cohesion, improved mood, etc.).

Key environmental pressures, such as disruption of ecosystems, loss of biodiversity, habitat destruction, pollution and climate change are driven by human behaviour and threaten the resilience of natural systems [99]. Many studies have shown that these changes have a direct effect on living conditions on earth and significant implications for the public health agenda worldwide [2,11]. Human health is profoundly dependent on undamaged natural environments and functioning ecosystems that provide life supporting services and resources [1]. It is well known that climate change caused by human interventions in biogeochemical cycles negatively impacts ecological balances and concomitant ecosystem services [100]. The Lancet Countdown on Health and Climate Change has recently outlined pathways by which climate change will affect human health worldwide, spanning from exacerbation of existing health problems to introduction of new threats [10]. Anthropogenic climate change also has the potential to affect ecosystem services in terms of immunological health provisioning and regulation, especially by impacting the occurrence and spread of infectious diseases [10,101]. Examples are a change in the geographic range and resulting rise of vector-borne diseases [102], ancient viruses emerging from thawing permafrost [103,104] or the increase in allergenic pollen [105]. Mitigating climate change might therefore yield considerable co-benefits for human health, and joined-up policy making could be seen as a great opportunity for enforcing global health priorities in the 21st century. This study may contribute to a novel, uncommon argumentation in future nature protection and climate change mitigation debates and encourage green policy measures that benefit human and planetary health alike. At large, this review supports the notion that the conservation and creation of healthy human habitats along with the promotion of a nature-connected lifestyle create a new opportunity to support immunological health provisioning.

## 7. Conclusions

This systematic review gathers promising evidence that nature exposure influences measurable immunological parameters in healthy individuals as well as in people suffering from acute or chronic inflammatory conditions, and that inhaling certain volatile natural compounds can have a beneficial effect on the elicited immune response. According to the synthesis of the studies included in this review, nature exposure supports immunological homeostasis and might offer promising strategies for therapeutic and preventive clinical use. However, there is a lack of studies that rigorously address questions of selectivity, effectivity or adverse effects deriving from nature exposure, let alone providing mechanistic pathway analyses or a solid calculation of effect sizes. This is highly necessary to guarantee outcome reproducibility and safety of nature-based therapies for future broader applications. There is a need for expanding the study field to a larger scale and bigger study cohorts, including different study populations and environments, a standardised control for confounders and environmental conditions as well as the use of more in-depth, state-of-the-art analytical methods and tools. This is essential to draw adequate conclusions and envision a future potential for nature exposure in immunological disease prevention or treatment.

## Figures and Tables

**Table 3:** Quality assessment of human intervention studies following the EPHPP tool.

								Overall Score	
		Selection Bias	Study Design	Con-founders	Blinding	Data Collection	Dropouts		Without Blinding *
Gaisberger et al.	2012	2	1	1	3	1	3	3	2
Grafetstätter et al.	2017	3	1	2	3	1	3	3	3
Han et al.	2016	2	1	1	3	1	3	3	2
Im et al.	2016	2	1	1	3	1	3	3	2
Jia et al.	2016	3	1	1	3	1	3	3	3
Kiecolt-Glaser et al.	2008	2	2	3	1	3	1	3	3
Kim et al.	2015	2	2	NA	3	1	1	2	1
Komori et al.	1995	3	2	2	3	1	3	3	3
Li et al.	2007	3	2	NA	3	1	2	3	2
Li et al.	2008a	3	2	NA	3	1	3	3	3
Li et al.	2008b	3	2	NA	3	1	2	3	2
Li et al.	2009	3	2	NA	3	1	2	3	2
Lyu et al.	2019	3	1	2	3	1	2	3	2
Mao et al.	2012a	3	1	1	3	1	2	3	2
Mao et al.	2012b	3	1	1	3	1	2	3	2
Mao et al.	2017	2	1	1	3	1	1	2	1
Mao et al.	2018	2	1	1	3	1	1	2	1
Seo et al.	2015	2	2	NA	3	1	2	2	1
Trellakis et al.	2012	2	2	NA	2	1	1	1	1
Tsao et al.	2018	3	2	1	3	1	3	3	3

1 = strong (green), 2 = moderate (yellow), 3 = weak (red), NA = not applicable (one-group studies). An alternative overall score excludes the “blinding” category (*).

**Table 4:** Quality assessment of animal intervention studies following the ARRIVE guidelines.

(a)											
		1	2	3	4	5	6	7	8	9	10
		Title	Abstract	Introduction		Methods					
				**Background**	**Objectives and Hypotheses**	**Ethical Statement**	**Study Design (Number of Experimental Groups, Blinding, Experimental Unit)**	**Experimental Procedure**	**Animal Details (Species, Sex, Age, Source, Weight)**	**Housing and Husbandry Conditions**	**Sample size Calculation (Number per Group, Number of Independent Replicates)**
Ahn et al.	2018a	2	1	1	1	1	3	1	1	1	2
Ahn et al.	2018b	2	1	1	1	2	2	1	2	1	2
Bastos et al.	2011	1	1	1	1	1	2	1	2	3	3
Bibi et al.	2015	1	2	1	1	2	2	1	2	1	2
Fujiwara et al.	1998	1	1	2	2	3	2	2	2	2	1
Hansen et al.	2013	2	1	1	2	1	2	1	1	1	2
Hansen et al.	2016	1	1	1	1	1	1	1	1	1	2
Hirota et al.	2012	1	2	2	3	2	1	1	1	1	2
Keinan et al.	2005	1	1	1	1	2	3	1	3	3	3
Kennedy-Feitosa et al.	2019	1	1	2	2	2	2	1	1	1	2
Lee et al.	2016	2	1	1	1	2	3	1	1	3	3
Nakamura et al.	2009	2	2	2	3	3	2	1	2	1	2
Yang et al.	2015	1	1	1	1	1	3	3	2	1	3
**(b)**											
		**11**	**12**	**13**	**14**	**15**	**16**	**17**	**18**	**19**	**20**
		**Methods**			**Results**				**Discussion**		
		**Allocation to Experimen-tal Groups (Randomi-sation, Matching)**	**Experi-mental Outcomes**	**Statistical Analysis**	**Baseline Data Monitoring**	**Number of Animals Analysed**	**Outcome Report and Estimation (Individual Values, Standard Deviation)**	**Adverse Events**	**Interpreta-tion and Limitations**	**Generalis-ability and Translational Relevance**	**Funding**
Ahn et al.	2018a	3	1	1	3	3	2	3	2	2	1
Ahn et al.	2018b	3	1	1	3	3	2	3	2	2	3
Bastos et al.	2011	3	1	1	3	3	2	3	2	2	2
Bibi et al.	2015	3	1	1	3	1	2	3	2	1	1
Fujiwara et al.	1998	3	3	2	3	3	2	3	2	2	3
Hansen et al.	2013	3	1	1	1	2	1	3	1	1	1
Hansen et al.	2016	2	1	1	1	1	2	3	1	1	1
Hirota et al.	2012	2	1	1	3	1	2	3	2	2	2
Keinan et al.	2005	3	2	2	3	3	2	3	3	2	3
Kennedy-Feitosa et al.	2019	3	1	1	3	1	2	3	1	1	1
Lee et al.	2016	3	1	1	3	3	2	3	2	2	1
Nakamura et al.	2009	3	1	1	3	1	2	3	2	2	3
Yang et al.	2015	3	1	1	3	3	2	3	2	2	1

1 = reported/strong (green), 2 = moderate (yellow), 3 = not reported/weak (red).

**Table 5:** Outcomes of human studies. Significances are given with *p* < 0.05, *p* < 0.01 and *p* < 0.001; NS = not significant, NA = not applicable. A non-significant trend is described as “Decrease/Increase, NS”. If significances are not given, it is described as “Decrease/Increase, NA”.

				Outcome								
Main Author	Year	Title	Measure	Intervention (Compared to Baseline)	*p*-Value	Control (Compared to Baseline)	*p*-Value	Comparison (Intervention Compared to Control)	*p*-Value	Conclusion		Comment
**Forest bathing**												
Han et al. [45]	2016	The effects of forest therapy on coping with chronic widespread pain	NK cell activity	Increase	*p <* 0.001	NS		Increase	NA	NK cell activity increases after a forest bathing trip in adults with chronic pain.		*Significant baseline differences between control and intervention group in NK cell activity.*
Im et al. [44]	2016	Comparison of effect of two-hour exposure to forest and urban environments on cytokine, anti-oxidant and stress levels in young adults	IL-6 level in serum					NS		Pro-inflammatory cytokine level (IL-8 and TNFα, but not IL-6) is reduced in healthy students after a forest bathing trip.		*No pre-intervention data shown.*
			IL-8 level in serum					Decrease	*p <* 0.001			
			TNFα level in serum					Decrease	*p <* 0.001			
			Glutathione peroxidase (GPx) level in serum					Increase	*p <* 0.05			
Jia et al. [46]	2016	Health effects of forest bathing trip on elderly patients with chronic obstructive pulmonary disease	CD8+ T cell number and %	NS		NS		NS		Forest bathing reduces pro-inflammatory cytokine levels, but not proportions of CD8+ T, NK or NKT cells or GrB expression in COPD patients.		*Result on perforin expression is questionable, since there is also an effect in control group.*
			NK cell number and % (CD3-CD56+)	NS		NS		NS				
			NKT cell number and % (CD3+CD56+)	NS		NS		NS				
			Perforin expression in CD8+ T cells (flow cytometry)	Decrease	*p <* 0.001	Decrease	NA	Decrease	*p <* 0.01			
			Perforin expression in NK cells (flow cytometry)	Decrease	*p <* 0.001	Decrease	NA	NS				
			Perforin expression in NKT cells (flow cytometry)	Decrease	*p <* 0.001	Decrease	NA	Decrease	*p <* 0.05			
			Granzyme B expression in CD8+ T cells (flow cytometry)	NS		NS		NS				
			Granzyme B expression in NK cells (flow cytometry)	NS		NS		NS				
			Granzyme B expression in NKT cells (flow cytometry)	NS		NS		NS				
			IL-6 level in serum	Decrease	*p <* 0.01	NS		Decrease	*p <* 0.05			
			IL-8 level in serum	Decrease	*p <* 0.05	NS		Decrease	*p <* 0.01			
			IFN-y level in serum	Decrease	*p <* 0.01	NS		Decrease	*p <* 0.05			
			IL-1b level in serum	NS		NS		Decrease	*p <* 0.05			
			CRP level in serum	NS		NS		Decrease	*p <* 0.05			
			TNFα level in serum	NS		NS		NS				
Kim et al. [52]	2015	Forest adjuvant anti-cancer therapy to enhance natural cytotoxicity in urban women with breast cancer: A preliminary prospective interventional study	NK cell number (CD3-CD56+)	Increase	*p <* 0.01					Forest therapy enhances natural cytotoxicity in breast cancer patients by increasing NK cells and cytotoxic mediators.		
			Perforin level in serum (ELISA)	Increase	*p <* 0.02							
			Granzyme B level in serum (ELISA)	Increase	*p <* 0.02							
Li et al. [53]	2007	Forest bathing enhances human natural killer activity and expression of anti-cancer proteins	NK cell number and % (CD16+)	Increase	*p <* 0.01					Forest bathing enhances NK cell activity and numbers in healthy male adults.		
			Cytolytic NK cell activity (Cr-release assay)	Increase	*p <* 0.01							
			% of T cells (CD3+)	Decrease	NA							
			% of perforin- expressing cells	Increase	*p <* 0.01							
			% of granzyme A/B- expressing cells	Increase	*p <* 0.01							
			% of granulysin-expressing cells	Increase	*p <* 0.01							
Li et al. [55]	2008a	A forest bathing trip increases human natural killer activity and expression of anti-cancer proteins in female subjects	NK cell number and % (CD16+)	Increase	*p <* 0.01					Forest bathing enhances NK cell activity and numbers in healthy female adults.		
			Cytolytic NK cell activity (Cr-release assay)	Increase	*p <* 0.01							
			% of T cells (CD3+)	Decrease	*p <* 0.05							
			% of perforin- expressing cells	Increase	*p <* 0.01							
			% of granzyme A/B- expressing cells	Increase	*p <* 0.01							
			% of granulysin- expressing cells	Increase	*p <* 0.01							
Li et al. [54]	2008b	Visiting a forest, but not a city, increases human natural killer activity and expression of anti-cancer proteins	NK cell number and % (CD16+)	Increase	*p <* 0.01	NS		Increase	*p <* 0.05	Forest bathing enhances NK cell activity and numbers in healthy adults.		
			Cytolytic NK cell activity (Cr-release assay)	Increase	*p <* 0.01	NS		Increase	*p <* 0.05			
			% of T cells (CD3+)	NS		NS						
			% of perforin- expressing cells	Increase	*p <* 0.01	NS						
			% of granzyme A/B- expressing cells	Increase	*p <* 0.01	NS						
			% of granulysin- expressing cells	Increase	*p <* 0.01	NS						
Lyu et al. [47]	2019	Benefits of a three-day bamboo forest therapy session on the psychophysiology and immune system responses of male college students	Cytolytic NK cell activity	Increase	*p <* 0.05	NS			NA	Forest bathing in a bamboo forest enhances NK cell activity and percentages in healthy adults.		
			% of NK cells (CD16+CD56+)	Increase	*p <* 0.05	NS			NA			
			Perforin level (ELISA)	Increase	*p <* 0.05	NS			NA			
			Granulysin level (ELISA)	NS		NS			NA			
			Granzyme A/B level (ELISA)	Increase	*p <* 0.05	NS			NA			
Mao et al. [50]	2012a	Effects of short-term forest bathing on human health in a broad-leaved evergreen forest in Zhejiang Province, China	IL-6 level in serum (radioimmunoassay)	NS		Increase	NA	Decrease	*p <* 0.05	Forest bathing decreases pro-inflammatory cytokine levels (IL-6 and TNFα) in healthy young adults but has no effect on immune cell distribution.		*Questionable effect (increase) in control group compared to baseline for IL-6 and TNFα levels.*
			TNFα level in serum (radioimmunoassay)	Decrease	NA	Increase	NA	Decrease	*p <* 0.05			
			HCRP level in serum					NS				
			% of B cells (CD5+CD19+)					Increase	*p <* 0.05			*No pre-intervention data shown for leukocyte distributions.*
			% of T cells (CD3+)					NS				
			% of Th cells (CD3+CD4+)					NS				
			% of cytotoxic T cells (CD3+CD8+)					NS				
			% of NK cells (CD3-CD16+CD56+)					NS				
Mao et al. [51]	2012b	Therapeutic effect of forest bathing on human hypertension in the elderly	IL-6 level in serum (radioimmunoassay)	Decrease	*p <* 0.05	NS		NS		Forest bathing decreases pro-inflammatory cytokine level (IL-6, but not TNFα) in elderly patients with hypertension.		
			TNFα level in serum (radioimmunoassay)	NS		NS		NS				
Mao et al. [49]	2017	The salutary influence of forest bathing on elderly patients with chronic heart failure	IL-6 level in serum (ELISA)	NS		NS		Decrease	*p <* 0.05	Forest bathing decreases pro-inflammatory cytokine level (IL-6, but not TNFα) in elderly patients with chronic heart failure.		*No effect in intervention group compared to baseline.*
			TNFα level in serum (ELISA)	NS		NS		NS				
			HCRP level in serum	NS		NS		NS				
Mao et al. [48]	2018	Additive benefits of twice forest bathing trips in elderly patients with chronic heart failure	IL-6 level in serum (ELISA)	NS		NS		NS		A second forest bathing trip further decreases pro-inflammatory cytokine level (TNFα, but not IL-6) in elderly patients with chronic heart failure.		
			TNFα level in serum (ELISA)	Decrease	*p <* 0.05	NS		Decrease	*p <* 0.05			
Seo et al. [56]	2015	Clinical and immunological effects of a forest trip in children with asthma and atopic dermatitis	*Asthma:*							Forest environment improves clinical symptoms in asthmatic children.		
			Forced vital capacity (spirometry, FCV)	Increase	*p <* 0.05							
			Fractional exhaled nitric oxide (FeNO)	Decrease	NA							
Seo et al. [56]	2015	Clinical and immunological effects of a forest trip in children with asthma and atopic dermatitis	*Atopic dermatitis:*							Forest environment improves clinical symptoms and has immunological effects in chronic allergic skin disease.		
			Atopic dermatitis index (SCORAD)	Decrease	NA							
			Thymus and activation-regulated chemokine/CCL17 level	NS								
			Macrophage-derived chemokine/CCL22 level	Decrease	*p <* 0.01							
Tsao et al. (forest workers) [57]	2018	Health effects of a forest environment on natural killer cells in humans: an observational pilot study	% of NK cells in blood (CD3-CD56+)					Increase	*p <* 0.05	Living in a forest environment increases NK cell percentage, but not the amount of activated NK cells.		
			% of activated NK cells in blood (CD3-CD56+CD69+)					NS				
Tsao et al. (forest bathing) [57]	2018	Health effects of a forest environment on natural killer cells in humans: an observational pilot study	% of NK cells in blood on d5	NS						Short-term forest trip enhances fraction of activated NK cells in healthy adults, and effect lasts for at least 4 days.		
			% of activated NK cells in blood on d5	Increase	*p <* 0.01							
			% of NK cells in blood on d9 (4 days after intervention)	NS								
			% of activated NK cells in blood on d9 (4 days after intervention)	Increase	*p <* 0.01							
**BVOC inhalation**												
Li et al. [58]	2009	Effect of phytoncide from trees on human natural killer cell function	Cytolytic NK cell activity (Cr-release assay)	Increase	*p <* 0.05					Phytoncide exposure enhances NK cell activity and % in healthy adults.		
			% of NK cells (CD16+)	Increase	*p <* 0.01							
			% of T cells (CD3+)	Decrease	*p <* 0.01							
			% of perforin- expressing cells	Increase	*p <* 0.05							
			% of granzyme A/B- expressing cells	Increase	*p <* 0.01 *p <* 0.05							
			% of granulysin- expressing cells	Increase	*p <* 0.05							
**Fragrance inhalation**												
Kiecolt-Glaser et al. [59]	2008	Olfactory influences on mood and autonomic, endocrine and immune function	Delayed hypersensitivity to candida (DTH)	Increase	NA	Increase	NA	Decrease	*p* = 0.02 (lavender) *p* = 0.06 (lemon)	Greater DTH response after water inhalation indicates better immune response than in fragrance groups, but no difference in cytokine levels detectable.		* Differing effects in blinded and informed groups for blastogenesis responses.
			PBL proliferation (blastogenesis)					NA*				
			IL-6 level in PBLs (ELISA)					NS				
			IL-10 level in PBLs (ELISA)					NS				
Komori et al. [60]	1995	Effects of citrus fragrance on immune function and depressive states	Deviation from normal CD4/CD8 ratio	Decrease	NA					Citrus fragrance has a beneficial effect on immune cell distribution in depressive patients and can reduce the dose antidepressants needed.		
			Deviation from normal NK cell activity (Cr-release assay)	Decrease	NA							
Trellakis et al. [61]	2012	Subconscious olfactory influences of stimulant and relaxant odors on immune function	IL-8 level in serum (ELISA)					NS		No significant effect of any stimulatory or relaxing fragrance exposure on immune parameters in healthy adults.		
			IL-6 level in serum (ELISA)					NS				
			TNFα level in serum (ELISA)					NS				
			CCL3 (MIP-1a) level in serum (ELISA)					NS				
			CCL4 (MIP-1b) level in serum (ELISA)					NS				
			CCL5 (RANTES) level in serum (ELISA)					NS				
			CXCL8 (IL-8) release by neutrophils					NS				
**Waterfall exposure**												
Gaisberger et al. [35]	2012	Effects of ionized waterfall aerosol on pediatric allergic asthma	IL-5 level in serum (ELISpot)	Decrease	*p <* 0.05	NS		Decrease	NS	Exposure to waterfalls reduces pro-inflammatory cytokines and allergic asthma symptoms, enhances lung function and induces Treg cells.		
			IL-10 level in serum (ELISpot)	Increase	*p <* 0.05	NS		Increase	NS			
			IL-13 level in serum (ELISpot)	Decrease	*p <* 0.01	Decrease	*p <* 0.01	NS				
			IL-10 expression (PCR)	Increase	NA	Increase	NA	NS				
			IL-13 expression (PCR)	Decrease	NA	NS	NA	Decrease	*p <* 0.05			
			IFNg expression (PCR)	Increase	NA	Increase	NA	NS				
			Treg cells (%)	Increase	*p <* 0.01	Increase	*p <* 0.05	NS				
			Eosinophilic cationic protein (ECP) levels in serum	Decrease	*p <* 0.05	NS		NS				
			Fractional exhaled nitric oxide (FeNO) at d20	Decrease	*p <* 0.001	Decrease	*p <* 0.001		NA			
			Fractional exhaled nitric oxide (FeNO) at d80	Decrease	*p <* 0.01	NS			NA			
			Peak expiratory flow rate (PEF)	Increase	*p <* 0.01	Increase	*p <* 0.01		NA			
			Other spirometric parameters (FEV, FEV%FVC, FEF25, FEF50, MMEF2575)	Increase	*p <* 0.05 *p <* 0.01	NS			NA			
Grafetstätter et al. [34]	2017	Does waterfall aerosol influence mucosal immunity and chronic stress? A randomized controlled clinical trial	IgA level in saliva (d6)	Increase	NA	Increase	NA	Increase	*p* = 0.001	Exposure to waterfalls activates the immune system and improves lung function.		
			IgA level in saliva (d66)	Increase	NA	NS		Increase	*p <* 0.05			
			Peak expiratory flow rate (PEF) (d6)	Increase	*p* = 0.023	Increase	NA	NS				

**Table 6:** Outcomes of animal studies. Significances are given with *p* < 0.05, *p* < 0.01 and *p* < 0.001; NS = not significant, NA=not applicable. A non-significant trend is described as “Decrease/Increase, NS”. If significances are not given, it is described as “Decrease/Increase, NA”.

				Outcome					
Main Author	Year	Title	Measure	Pre-treatment	*p*-Value	Intervention (Compared to Pre-treatment)	*p*-Value	Conclusion	Comment
**BVOC inhalation**									
Ahn et al. [62]	2018a	Alleviation effects of natural volatile organic compounds from Pinus densiflora and Chamaecyparis obtuda on systemic and pulmonary inflammation	IgE level in serum (ELISA)	Increase	*p <* 0.05	Decrease	*p <* 0.05	BVOCs (VOCCo, VOCPd) excert anti-inflammatory effects in mice.	
			Prostaglandin E2 (PGE2) level in serum (ELISA)	Increase	*p <* 0.05	Decrease	*p <* 0.05		
			COX-2 mRNA expression in PBMCs	Increase	*p <* 0.05	Decrease	*p <* 0.05		
			TNFα mRNA expression in PBMCs	Increase	*p <* 0.05	Decrease	*p <* 0.05		
			IL-1b mRNA expression in PBMCs	Increase	*p <* 0.05	Decrease	*p <* 0.05		
			IL-13 mRNA expression in PBMCs	Increase	*p <* 0.05	Decrease	*p <* 0.05		
			COX-2 mRNA expression in lung tissue	Increase	*p <* 0.05	Decrease	*p <* 0.05		
			NF-kB mRNA expression in lung tissue	Increase	*p <* 0.05	Decrease	*p <* 0.05		
			TNFα mRNA expression in lung tissue	Increase	*p <* 0.05	NS			
			COX-2, NF-kB, IL-1b, TNFα, IL-13 mRNA in BALF cells	Increase	*p <* 0.05	Decrease	*p <* 0.05		
Ahn et al. [63]	2018b	Anti-asthmatic effects of volatile organic compounds from Chamaecyparis obtusa, Pinus densiflora, Pinus koraiensis, and Larix kaempferi wood panels	Thickening of bronchiolar wall (hypertrophy)	Increase	NA	Decrease	NA	BVOCs (VOCCo, VOCPd, VOCPk, VOCLk) excert anti-allergic effects in asthmatic mice.	
			IL-4 level in serum (ELISA)	Increase	*p <* 0.05	Decrease	*p <* 0.05		
			TNFα level in serum (ELISA)	Increase	*p <* 0.05	Decrease (C. obtusa)	*p <* 0.05		
			IL-4 mRNA expression in bronchioles	Increase	*p <* 0.05	Decrease	*p <* 0.05		
			IL-5 mRNA expression in bronchioles	Increase	*p <* 0.05	NS			
			IL-9 mRNA expression in bronchioles	Increase	*p <* 0.05	Decrease (C. obtusa)	*p <* 0.05		
			IL-13 mRNA expression in bronchioles	Increase	*p <* 0.05	Decrease	*p <* 0.05		
Yang et al. [64]	2015	Estimation of the environmental effect of natural volatile organic compounds from Chamaecyparis obtusa and their effect on atopic dermatitis-like skin lesions in mice	IgE level in serum	Increase	*p <* 0.05	Decrease	*p <* 0.05	Exposure to BVOCs (C. obtusa) ameliorates inflammatory skin reactions in mice with atopic dermatitis.	
			Mast cell infiltration into skin lesions	Increase	*p <* 0.05	Decrease	*p <* 0.05		
			IL-1b mRNA expression in skin lesions	Increase	*p <* 0.05	Decrease	*p <* 0.05		
			IL-6 mRNA expression in skin lesions	Increase	*p <* 0.05	Decrease	*p <* 0.05		
**Eucalyptol inhalation**									
Bastos et al. [74]	2011	Inhaled 1,8-Cineole reduces inflammatory parameters in airways of ovalbumin-challenged guinea pigs	TNFα level in BALF (ELISA)	Increase	*p <* 0.05	Decrease	NS	Eucalyptol (1,8-cineol) inhibits antigen-induced airway inflammation in guinea pigs.	
			IL-1b level in BALF (ELISA)	Increase	*p <* 0.05	Decrease	NS		
			IL-10 level in BALF (ELISA)	Decrease	*p <* 0.05	Increase	NS		
			Leukocyte number in BALF (eosinophils and neutrophils)	Increase	*p <* 0.05	Decrease	*p <* 0.05		
			MPO activity	Increase	*p <* 0.05	Decrease	*p <* 0.05		
Kennedy-Feitosa et al. [65]	2019	Eucalyptol promotes lung repair in mice following cigarette smoke-induced emphysema	TNFα level in lung tissue	Increase	*p <* 0.01	Decrease	*p <* 0.01	Eucalyptol reduces pro-inflammatory cytokines and neutrophil activation marker (MPO) after lung damage by cigarette smoke.	
			IL-1b level in lung tissue	Increase	*p <* 0.01	Decrease	*p <* 0.05		
			IL-6 level in lung tissue	Increase	*p <* 0.01	Decrease	*p <* 0.01		
			TGFß-1 level in lung tissue	Increase	*p <* 0.05	Decrease	*p <* 0.05		
			MPO activity in lung tissue	Increase	*p <* 0.01	Decrease	*p <* 0.05		
Lee et al. [66]	2016	Effect of 1,8-cineol in *Dermatophagiodes pteronyssinus*-stimulated bronchial epithelial cells and mouse model of asthma	IL-4 level in BALF (ELISA)	Increase	*p <* 0.01	Decrease	*p <* 0.05	Eucalyptol reduces pro-inflammatory cytokine expression (IL-4, IL-13, IL-17A) in house dust mite-allergic/asthmatic mice.	
			IL-13 level in BALF (ELISA)	Increase	*p <* 0.05	Decrease	*p <* 0.05		
			IL-17A level in BALF (ELISA)	Increase	*p <* 0.05	Decrease	*p <* 0.05		
			Neutrophil number in BALF	Increase	*p <* 0.05	Decrease	*p <* 0.05		
			Eosinophil number in BALF	Increase	*p <* 0.05	Decrease	*p <* 0.05		
			Lymphocyte number in BALF	Increase	*p <* 0.05	Decrease	*p <* 0.05		
			Der p-specific IgG1 in serum (ELISA)	Increase	*p <* 0.01	Decrease	*p <* 0.05		
			Airway restriction (Penh)	Increase	*p <* 0.01	Decrease	*p <* 0.05		
**Limonene inhalation**									
Bibi et al. [67]	2015	Treatment of asthma by an ozone scavenger in a mouse model	Aldehyde (ozone oxydation product) levels in BALF	Increase	NA	Decrease	NA	Prophylactic limonene inhalation protects against allergic asthma in mice.	
			Aldehyde (ozone oxydation product) levels in lung tissue	Increase	NA	Decrease	NA		
			Aldehyde (ozone oxydation product) levels in spleen	Increase	NA	Decrease	NA		
			Neutrophil number in BALF	Increase	NA	Decrease	*p <* 0.05		
			Eosinophil number in BALF	Increase	NA	NS			
			Infiltration of inflammatory cells into lung tissue	Increase	NA	Decrease	NA		
Hirota et al. [68]	2012	Limonene inhalation reduces allergic airway inflammation in *Dermatophagoides farinae*-treated mice	Der f-specific IgG in serum (ELISA)	Increase	*p <* 0.001	Decrease	*p <* 0.01	Limonene reduces pro-inflammatory cytokines and cell numbers in mice pre-sensitized to house dust mite allergen.	
			Total IgE in serum (ELISA)	Increase	NS	Decrease	NS		
			Eosinophil number in BALF	Increase	*p <* 0.001	Decrease	*p <* 0.001		
			Lymphocyte number in BALF	Increase	*p <* 0.001	Decrease	*p <* 0.05		
			Neutrophil number in BALF	Increase	*p <* 0.001	Decrease	*p <* 0.05		
			Macrophage number in BALF	Increase	*p <* 0.001	Decrease	*p <* 0.05		
			IL-5 level in BALF	Increase	*p <* 0.001	Decrease	*p <* 0.001		
			IL-13 level in BALF	Increase	*p <* 0.001	Decrease	*p <* 0.001		
			Eotaxin level in BALF	Increase	*p <* 0.001	Decrease	*p <* 0.001		
			MCP-1 level in BALF	Increase	*p <* 0.001	Decrease	*p <* 0.001		
			TGF-b level in BALF	Increase	*p <* 0.001	Decrease	*p <* 0.05		
			IFNy level in BALF	Decrease	*p <* 0.01	Increase	*p <* 0.05		
			Bronchorestriction with Ach	Increase	*p <* 0.01	Decrease	*p <* 0.01		
Keinan et al. [73]	2005	Natural ozone scavenger prevents asthma in sensitized rats				*Limonene* *inhalation:*		Limonene reduces inflammatory cell infiltrates into lung tissue and improves airway restriction in lungs of rats with allergic asthma.	*No significances provided in graphical outcome report*
			Inflammatory cell infiltrates	Increase	NA	Decrease	NA		
			Airway restriction (Penh)	Increase	NA	Decrease	NA		
						*Eucalyptol* *inhalation:*		Eucalyptol reduces inflammatory cell infiltrates into lung tissue, but to a lesser extent than limonene, but does not improve airway restriction.	
			Inflammatory cell infiltrates	Increase	NA	Decrease	NA		
			Airway restriction (Penh)	Increase	NA	NS			
**Limonene/ozone inhalation**									
Hansen et al. [69]	2013	Adjuvant and inflammatory effects in mice after subchronic inhalation of allergen and ozone-initiated limonene reaction products				*Limonene* *inhalation:*		Limonene inhalation has no significant effect on inflammatory response in pre-sensitized mice.	*No naive (baseline before OVA-sensitisation) data shown.*
			OVA-specific IgE in serum (ELISA)	Increase	NA	NS			
			Eosinophil number in BALF	NS		NS			
			Lymphocyte number in BALF	Increase	NA	NS			
			Neutrophil number in BALF	Increase	NA	NS			
			Macrophage number in BALF	Increase	NA	NS			
				*Ozone* *inhalation:*		*Limonene +* *ozone inhalation:*		Limonene/ozone mixture reduces allergen-specific reactions in pre-sensitized mice.	*No naive (baseline before OVA-sensitisation) data shown.*
			OVA-specific IgE in serum (ELISA)	Increase	NA	Increase	*p <* 0.05		
			Eosinophil number in BALF	Increase	NA	Decrease	*p <* 0.05		
			Lymphocyte number in BALF	Increase	NA	Decrease	NS		
			Neutrophil number in BALF	Increase	NA	Decrease	*p <* 0.05		
			Macrophage number in BALF	Increase	NA	NS			
Hansen et al. [70]	2016	Limonene and its ozone-initiated reaction products attenuate allergic lung inflammation in mice		*Air* *inhalation:*		*Limonene* *inhalation:*		Limonene potentially reduces airway inflammation in allergic mice, however no significances given.	*Unclear graphical and verbal description of outcomes and signifi-cances.* *No OVA-only control.* *No comparisons between relevant groups.*
			OVA-specific IgE in serum (ELISA)	Increase	NA	Decrease	NA		
			OVA-specific IgG1 in serum (ELISA)	Increase	NA	NS			
			Eosinophil number in BALF	Increase	NA	NS			
			Lymphocyte number in BALF	Increase	NA	Increase	NA		
			Neutrophil number in BALF	Increase	NA	Decrease	NA		
			Macrophage number in BALF	Increase	NA	Decrease	NA		
			IL-5 expression in BALF	Increase	NA	Decrease	NA		
				*Ozone* *inhalation:*		*Limonene +* *ozone inhalation:*		Limonene/ozone mixture potentially attenuates allergic inflammation and ozone-induced pulmonary irritation in allergic mice.	*Unclear graphical and verbal description of outcomes and signifi-cances.* *No OVA-only control.* *No comparisons between relevant groups.*
			OVA-specific IgE in serum (ELISA)	Increase	NA	Decrease	NA		
			OVA-specific IgG1 in serum (ELISA)	Increase	NA	Increase	NA		
			Eosinophil number in BALF	Increase	NA	Decrease	*p <* 0.05		
			Lymphocyte number in BALF	Increase	NA	Decrease	NA		
			Neutrophil number in BALF	NS		NA			
			Macrophage number in BALF	Increase	NA	Decrease	NA		
			IL-5 expression in BALF	Increase	NA	Decrease	NA		
**Linalool inhalation**									
Nakamura et al. [72]	2009	Stress repression in restrained rats by R-(-)-linalool inhalation and gene expression profiling of their whole blood cells	% of neutrophils	Increase	*p <* 0.05	Decrease	NA	Linalool inhalation reverts stress-induced changes in neutrophil and lymphocyte fractions.	
			% of lymphocytes	Decrease	*p <* 0.05	Increase	NA		
**Fragrance inhalation**									
Fujiwara et al. [71]	1998	Effects of a long-term inhalation of fragrances on the stress-induced immunosuppression in mice	*Number of plaque forming cells (PFC)* *in spleen:*					Exposure to natural fragrances reverses stress-induced thymic involution and activates the immune system.	
			Lemon inhalation	Decrease	*p <* 0.05	Increase	*p <* 0.05		
			Oakmoss inhalation	Decrease	*p <* 0.05	Increase	*p <* 0.05		
			Labdanum inhalation	Decrease	*p <* 0.05	Increase	*p <* 0.05		
			Tuberose inhalation	Decrease	*p <* 0.05	Increase	*p <* 0.05		
			*Thymic weight:*						
			Lemon inhalation	Decrease	*p <* 0.05	Increase	*p <* 0.05		
			Oakmoss inhalation	Decrease	*p <* 0.05	Increase	*p <* 0.05		
			Labdanum inhalation	Decrease	*p <* 0.05	Increase	*p <* 0.05		
			Tuberose inhalation	Decrease	*p <* 0.05	Increase	*p <* 0.05		

## Data Availability

We have no archived datasets, since we did not produce any primary data in this review.

## Supplementary Materials

The following are available online at https://www.mdpi.com/1660-4601/18/4/1416/s1, Table S1: Risk of bias assessment of animal intervention studies following the SYRCLE guidelines.
