# Supplementary material for: Nature Exposure and Its Effects on Immune System Functioning: A Systematic Review
Source: Int J Environ Res Public Health. 2021 Feb 3;18(4):1416. doi: 10.3390/ijerph18041416 (PMC7913501; doi:10.3390/ijerph18041416)
Supplement: Supplementary file 1 [file ijerph-18-01416-s001.zip › Supplementary materials_210106.docx]

Supplementary Materials

## Scoring criteria in the EPHPP quality assessment of human studies

The EPHPP quality assessment tool provides a manual of suggested scoring criteria, which were followed wherever possible. Individual interpretations of scoring criteria are described below.

The selection bias category describes whether the method of participant selection was representative of the target population. Studies which used self-referred samples or recruited subjects from a non-representative group, e.g. specific professionals, had a high risk of selection bias and got a “weak” rating. If subjects were recruited from a representative source, e.g. a clinic, and/or selected from a comprehensive list of exclusion criteria, studies were rated “moderate”. No studies randomly selected participants from a list of individuals in the target population (“strong”). In the study design category, RCTs and CCTs (10 out of 20) were rated “strong”. All other studies were carried out as one-group pre-post intervention studies and rated “moderate”. Due to a lack of control groups the description of potential confounders was not possible, therefore this parameter was left out in these studies (NA = not applicable). Most of the remaining studies controlled for confounders such as sex, age, health status and other pre-intervention measures and were thus rated “strong”, while three studies only controlled for some confounders (“moderate”) and one study for none (“weak”). Most studies did not mention anything on blinding (“weak”). One study correctly reported blinding of outcome assessors and participants and was rated “strong”. Another study described blinding of participants but not outcome assessors and therefore was rated “moderate”. The data collection category assesses validity and reliability of data collection tools, which we rated “strong” in all studies except one. Only one study used inadequate and out-dated methods for appropriate outcome reporting of immunological results. Concerning withdrawals and dropouts, many studies did not explicitly report this parameter. The reason might be that no dropouts or withdrawals occurred due to a low number of participants. Therefore, we rated this parameter with “moderate” if the number of analysed individuals was given in the results and no dropouts had occurred. Studies which also failed to report any final numbers of participants were rated “weak”. A “strong” rating was given if either dropout numbers or reasons for exclusion were given.

1. Scoring criteria in the ARRIVE quality assessment of animal studies

The ARRIVE guidelines provide recommendations for correct reporting and assessment of animal studies in 20 categories, which were followed wherever possible. Individual interpretations of scoring criteria are described below.

All studies provided a good to moderate description of the content in title and abstract, briefly outlining background, objectives, methods, findings and conclusions of the study. The introduction section had to elaborate on the scientific background and include sufficient description of objectives and hypotheses being tested, which most of the studies did. Two studies failed to provide a clear description of study outcomes in the introduction section.

The requirements concerning the methods section were more specific and included several categories. In category 5, ethical statements were rated “strong” if national or international guidelines were followed, “moderate” if guidelines of the local animal facility were followed and “weak” if no guidelines were mentioned. In category 6, assessing the quality of study design, two studies accurately described the groups and numbers of animals used and provided a graphical overview of the study protocol as well as additional group allocation information, which was defined as “strong” study design. The rest of the studies reported either insufficient (“moderate”) or no (“weak”) information. In category 7, 8 and 9, the majority of studies presented detailed information on experimental procedures (what, how, concentration, duration, sacrifice method), animals (species, gender, age, source and weight) and housing conditions (specific pathogen free [SPF] or fixed environmental conditions such as temperature, circadian light-dark rhythm, bedding material). Studies clearly describing experimental sensitisation and intervention procedures were rated “strong”, if one parameter was missing with “moderate” and if no clear description was provided with “weak” in category 7. Studies providing a full or partial description of animal characteristics were rated “strong” or “moderate” in category 8, respectively. One study did not mention any animal details in the methods section and therefore got a “weak” rating. Three studies did not provide any details on housing conditions, resulting in “weak” ratings in category 9. In category 10 and 11, no study provided a calculation of sample sizes or appropriately described the method of allocation to experimental groups. However, one study correctly reported the number of animals used in each group as well as the number of independent replicates, which we assessed as “strong” in category 10. The majority of studies only mentioned the number of animals per group without providing specific information on experiment repetitions and were assessed with “moderate”. Studies not mentioning sample sizes got a “weak” assessment. In category 11, two studies were rated “moderate” since they used the term “randomly” to describe the method of allocation, but did not provide any details. In category 12 and 13, almost all studies provided sufficient information regarding the experimental outcomes and also included a description of the statistical analysis. Only two studies did not define primary and secondary outcomes, or did so vaguely, and failed to describe the method of statistical analysis (e.g. only mentioned briefly in the running text) and were therefore rated “moderate” or “weak”.

Most reporting shortcomings were detected in the results section. Only two studies gave information on baseline data in a way that specific animal characteristics such as body weight were monitored throughout the experiment, thereby receiving a “strong” rating in category 14. In category 15, seven studies did not report any information on the numbers of animals included in the final analysis or refer to dropout rates or reasons for exclusions (“weak”). Studies that reported the numbers included in their final analysis (in text or in figure legends) and where the final numbers matched the initial sample sizes were assessed with “strong”, while studies that reported differing numbers but no exclusion reasons were assessed with “moderate”. In category 16, all studies included a measure of precision (e.g. bars representing standard deviations) in their outcome report. However, only one study also provided data on the number of individual data points within one group, which should be considered the gold standard of outcome reporting. Thus, only one study got a “strong” outcome report rating, while the rest was rated “moderate”. No study reported if adverse effects had occurred; thus all studies got a “weak” rating in category 17.

Regarding the discussion section, most studies interpreted the implications of their findings within the current scientific literature; however, many failed to elaborate on study limitations concerning methodological imprecision, potential sources of bias or limitations of the chosen animal model or study design, amongst others, and were rated “moderate”. Only three studies included all these aspects in their discussion (“strong”) and one study failed to appropriately discuss any of these points (“weak”). Category 19 evaluated whether the discussion also commented on generalisability of study outcomes, i.e. whether and how the findings are translatable into the human system or relevant for human health. Many studies did not discuss translational relevance sufficiently, and were assessed with “moderate”, but four studies were rated “strong” since they explicitly discussed this issue. The last category evaluated the correct listing of funding sources including grant numbers as well as indicating or neglecting conflict of interests. Studies that included all of these points got a “strong” rating, whereas studies that mentioned either funding or made a conflict of interest statement got a “moderate” rating. If none of these were mentioned, studies were rated “weak”.

## Scoring criteria in the SYRCLE quality assessment of animal studies

As a second, supplementary quality assessment for animal studies, SYRCLE’s risk of bias tool was used to assess potential biases within 10 categories.

Many studies did not adequately report measures taken to reduce the risk of bias in several categories, which resulted in a high amount of “unclear” ratings for most indicators. Thus, none of the included studies reported how the sequence of allocating animals to experimental groups was generated (category 1), whether this allocation was adequately concealed so that investigators could not influence the group allocation i.e. by third-party coding or randomisation (category 3), whether investigators or caregivers were blinded from knowledge of the type of intervention each animal got (category 5), whether and how animals were randomly selected for outcome assessment (category 6) and whether the outcome assessor was blinded to knowledge of intervention i.e. during statistical analysis of results (category 7). Therefore, “unclear” ratings were given in all of these categories.

The majority of studies did not provide detailed information on whether the baseline characteristics were similar between groups before the experiment (category 2), and if not, whether the results were adjusted for confounders, which led to an overall “unclear” rating of most studies. As observed in the ARRIVE assessment scheme, only two studies reported monitoring of body weight during the experiment and were assessed with a “low” risk of bias in this category. One study failed to report any baseline data of the used animals, which resulted in a “high” risk of bias.

Concerning the question of random housing (category 4), the fact that most experimental setups aimed at investigating either long-term airborne exposures or volatile substances coming from housing conditions made it impossible to hold animals from the intervention and control groups in the same cage throughout the experiment. Therefore, we chose to refine the question in a way that fixed (e.g. SPF) or controlled/monitored conditions in the animal room resulted in a “low” risk of bias, since this makes it unlikely that the outcome is influenced by the housing conditions. “NA” was given for four studies that explicitly investigated housing conditions using different wood beddings or panels, due to their experimental question.

The question of attrition bias (category 8) asks whether all animals were included in the study and, if not, whether incomplete data were adequately addressed. Again, many studies failed to provide the final number of animals included in the results (see also ARRIVE assessment), resulting in an “unclear” attrition bias. Two studies did not provide the total number of animals used in the experiment, which we assessed with a “high” risk of bias. Five studies reported concordant numbers of animals included in experiment and results, which led to a “low” risk of bias assessment.

The majority of included studies were free from selective outcome reporting bias (category 9), since they included all results mentioned in the methods section and provided a clear (mostly graphical) depiction of the outcomes. However, two studies did not include controls or comparisons between relevant groups, which made it hard to conclude on the actual outcomes and findings of the study. Moreover, one of these studies also provided unclear and non-reproducible significance calculations, which did not match between graphical illustration and text. Therefore, these two studies were assessed with a “high” risk of reporting bias. One study did not include any analysis of significances, leading to an “unclear” rating.

Other sources of bias (category 10) include a lack in funding information, missing conflict of interest statements or design-specific risks of biases, which resulted in high risks of bias in six studies. All results of the SYRCLE risk of bias assessment are illustrated in Supplementary Table 1.


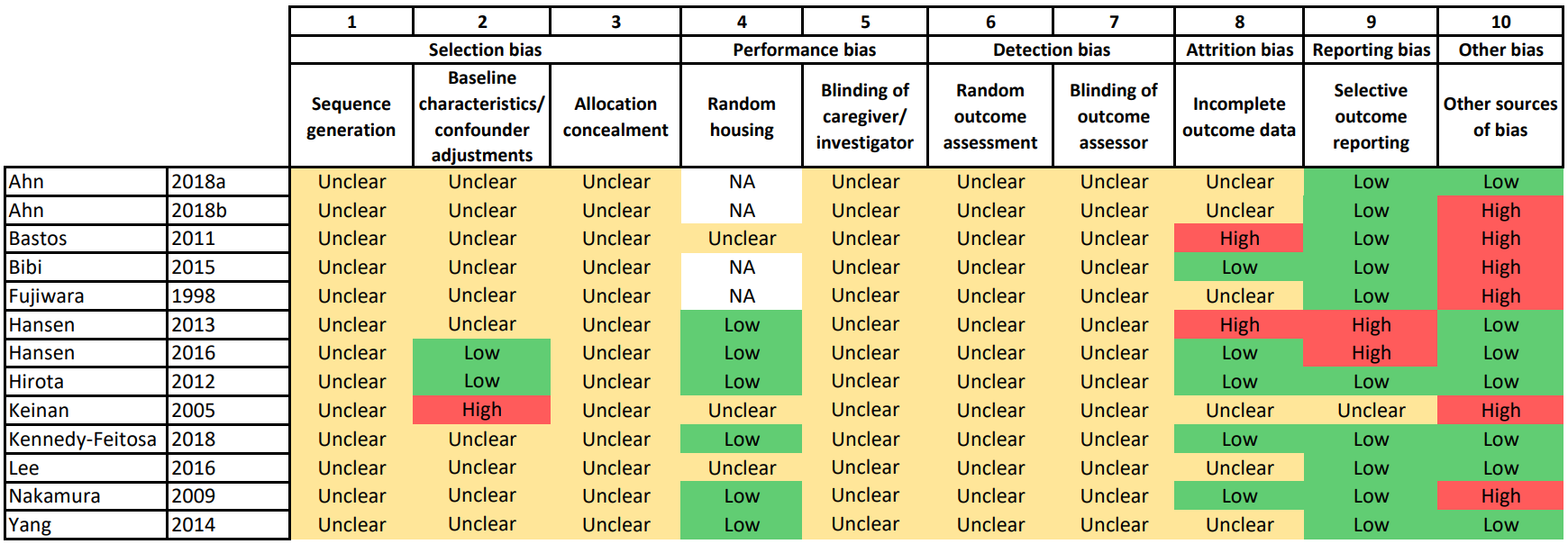


**Supplementary Table 1:** Risk of bias assessment of animal intervention studies following the SYRCLE guidelines and scoring scheme with low, high or unclear risk of bias. NA = not applicable

1. Scoring criteria for the use of valid and reliable methods

While most of the more recent studies used adequate and comparable methods to measure immune responses, older studies tended to use more diverse methods and less exact instrumental technologies. If the methods were adequate and reliable at the time the study was carried out, the quality of the methodological approach was positively assessed [57]. If the methods used were not adequate to address the specific question at the time the study was released, or if the methods did not meet the criteria of reliability, the methodological approach of a study was assessed as weak. Measuring the number of plaque-forming cells [68], the weight of the thymus [68], cellular proliferation rate/blastogenesis [56] or a delayed hypersensitivity to candida infection [56] were considered inadequate or not sufficient for assessing the strength of an immune response. Nevertheless, the majority of studies used up-to-date technologies such as flow cytometric (FACS) measurements and enzyme-linked immunosorbent assays (ELISA) that can accurately assess cellular ratios and percentages as well as cytokine and chemokine levels in a wide range of physiological fluid and tissue samples [103], [104]. These methods are, together with the application of clinical disease scores from tissue histology or organ function measurements (e.g. lung spirometry, skin reactions …), today widely used in immunological research and constitute state-of-the-art technologies for addressing questions on immune functioning.
