# Supplementary material for: Nature Exposure and Its Effects on Immune System Functioning: A Systematic Review
Source: Int J Environ Res Public Health. 2021 Feb 3;18(4):1416. doi: 10.3390/ijerph18041416 (PMC7913501; doi:10.3390/ijerph18041416)
Supplement: Supplementary file 1 [file ijerph-18-01416-s001.zip › Supplementary Table_SYRCLE assessment.docx]

|  |  | **1** | **2** | **3** | **4** | **5** | **6** | **7** | **8** | **9** | **10** |
| --- | --- | --- | --- | --- | --- | --- | --- | --- | --- | --- | --- |
|  |  | **Selection bias** | | | **Performance bias** | | **Detection bias** | | **Attrition bias** | **Reporting bias** | **Other bias** |
|  |  | **Sequence generation** | **Baseline characteristics/ confounder adjustments** | **Allocation concealment** | **Random housing** | **Blinding of caregiver/ investigator** | **Random outcome assessment** | **Blinding of outcome assessor** | **Incomplete outcome data** | **Selective outcome reporting** | **Other sources of bias** |
| Ahn et al. | 2018a | Unclear | Unclear | Unclear | NA | Unclear | Unclear | Unclear | Unclear | Low | Low |
| Ahn et al. | 2018b | Unclear | Unclear | Unclear | NA | Unclear | Unclear | Unclear | Unclear | Low | High |
| Bastos et al. | 2011 | Unclear | Unclear | Unclear | Unclear | Unclear | Unclear | Unclear | High | Low | High |
| Bibi et al. | 2015 | Unclear | Unclear | Unclear | NA | Unclear | Unclear | Unclear | Low | Low | High |
| Fujiwara et al. | 1998 | Unclear | Unclear | Unclear | NA | Unclear | Unclear | Unclear | Unclear | Low | High |
| Hansen et al. | 2013 | Unclear | Unclear | Unclear | Low | Unclear | Unclear | Unclear | High | High | Low |
| Hansen et al. | 2016 | Unclear | Low | Unclear | Low | Unclear | Unclear | Unclear | Low | High | Low |
| Hirota et al. | 2012 | Unclear | Low | Unclear | Low | Unclear | Unclear | Unclear | Low | Low | Low |
| Keinan et al. | 2005 | Unclear | High | Unclear | Unclear | Unclear | Unclear | Unclear | Unclear | Unclear | High |
| Kennedy-Feitosa et al. | 2019 | Unclear | Unclear | Unclear | Low | Unclear | Unclear | Unclear | Low | Low | Low |
| Lee et al. | 2016 | Unclear | Unclear | Unclear | Unclear | Unclear | Unclear | Unclear | Unclear | Low | Low |
| Nakamura et al. | 2009 | Unclear | Unclear | Unclear | Low | Unclear | Unclear | Unclear | Low | Low | High |
| Yang et al. | 2015 | Unclear | Unclear | Unclear | Low | Unclear | Unclear | Unclear | Unclear | Low | Low |
